# Role of complementary-sense genes and intergenic region of beet curly top virus in intermolecular recombination frequency upon local infection in plants

**DOI:** 10.1128/jvi.00016-25

**Published:** 2025-07-08

**Authors:** Omid Eini, Zahra Shoaei, Nadine Schumann, Mark Varrelmann

**Affiliations:** 1 Department of Phytopathology, Institute for Sugar Beet Research, Göttingen, Germany; 2 KWS SAAT SE & Co KGaA 39243 , Einbeck, Germany; Iowa State University, Ames, Iowa, USA

**Keywords:** plant virus, DNA recombination, geminivirus, protoplast, betasatellite, sugar beet

## Abstract

Intermolecular recombination is a critical process in the evolution of plant viruses, including geminiviruses. In this study, we investigated the impact of geminiviral factors on the homologous recombination frequency (HRF) in a model system involving replicons of the beet curly top virus (BCTVRepl) and the cotton leaf curl Multan betasatellite (CLCuMB), which lacks the β*C1* gene. BCTVRepl-cGFP and CLCuMB-nGFP replicated together and exhibited intermolecular recombination, which was monitored by reconstituted GFP fluorescence. Mutation of the *C2, C3,* and *C4* genes in BCTVRepl-cGFP led to a significant decrease in HRF. Accumulation of BCTVRepl-cGFP was reduced in the *C3* mutant but not in the *C2* and *C4* mutants. These findings suggest that *C4* and, to a greater extent, *C2* play a role in homologous recombination (HR) between BCTV and CLCuMB replicons. Furthermore, the BCTV large intergenic region (LIR) sequence to which the C1 (Rep) protein binds to initiate viral replication was introduced into the nGFP construct (referred to as LnGFP). This resulted in a low level of HR (5%) compared to the control nGFP construct, where HR did not occur. Subsequent mutational analysis of the iteron and stem-loop sequences within the LIR of LnGFP revealed the role of these sequences and the C1 (Rep) in the process of DNA recombination. This study presents a new model for assessing HRF in BCTV, which can be applied to other geminiviruses. It also highlights the roles of the *C2, C4, C1,* and LIR structural sequences in intermolecular recombination and replication in the BCTV replicon.

**IMPORTANCE:**

Intermolecular recombination is a critical process in the evolution, adaptability, and pathogenicity of plant viruses, including geminiviruses. This study developed a visible and quantifiable system to measure the frequency of homologous recombination between beet curly top virus (BCTV) and cotton leaf curl Multan betasatellite (CLCuMB) replicons based on the reconstitution of GFP fluorescence. Furthermore, mutation analysis of complementary-sense genes in the BCTV replicon indicated that *C4* and, to a greater extent, *C2* play a role in the recombination between the BCTV and CLCuMB replicons. Subsequent mutational analysis of the iteron and stem-loop sequences within the large intergenic region of BCTV revealed the possible role of these structural sequences in the recombination process.

## INTRODUCTION

Geminiviruses (family *Geminiviridae*) are circular single-stranded DNA (ssDNA) viruses that rely on host DNA polymerase for their replication (1). These viruses have small DNA genomes with limited coding capacities. They interact with a wide range of plant proteins and pathways during their infection cycle. Geminiviruses replicate by rolling circle replication (2) and also recombination-dependent replication (RDR) (3–5). Furthermore, geminiviruses reprogram the cell cycle of infected cells to induce the replication of both viral and plant chromosomal DNA (6). They also activate the host DNA repair machinery, promote somatic homologous recombination (HR) (7, 8), which may induce the RDR mechanism. These interactions have been shown to contribute to viral DNA replication and infection. However, the role of other geminiviral proteins in promoting HR remains to be elucidated.

The family *Geminiviridae* comprises 15 genera (https://ictv.global/taxonomy) including the genus *Curtovirus* which includes viruses with monopartite genomes, whereas members of the genus *Begomovirus* contain mono- or bipartite genomes (9). Beet curly top virus (BCTV) is a member of the genus *Curtovirus*, whose members have monopartite genomes of 2.8–3 kb in size that encode up to seven genes. Three of these genes are arranged in the virion sense, including the coat protein gene (CP, *V1*), which also functions as a nuclear shuttle protein for monopartite viruses (10), a regulatory gene (Reg, *V2*), which regulates the accumulation of ssDNA and double-stranded DNA (dsDNA) during the viral replication process (11), and a movement protein gene (MP, *V3*). The other four genes are arranged in the complementary-sense orientation and include replication-associated protein (Rep, *C1*) which is highly conserved across the *Geminiviridae* family (12) and has multiple roles in DNA replication, transcription of viral genes, and suppression of host defense responses (13), a gene that expresses a protein with RNA silencing suppressor functions (SS, *C2*) (14), replication enhancer (REn, *C3*) gene, and symptom determinant gene (SD*, C4*) (15, 16).

The genome of curtoviruses also contains a large intergenic region (LIR) that includes a sequence capable of forming a stem-loop structure with a universally conserved nonanucleotide motif (TAATATT/AC). This motif is common to all geminiviruses and contains the initiation site for rolling circle replication (17). The LIR also contains high-affinity binding sites for the Rep protein known as direct repeats or iterons. Binding of the Rep protein to these iterons is essential for viral replication (18). The strain-specific element in BCTV DNA replication has been shown to map to the directly repeated motif (iterons) in the LIR (19). Rep initiates rolling circle replication by binding to these sites within the LIR, creating a single-strand nick within the nonanucleotide motif located at the apex of a conserved stem-loop (hairpin) structure.

Betasatellites are defined as small circular subviral ssDNA molecules with a size of 1.5 kb. These molecules depend on a helper geminivirus for encapsidation, replication, movement, and vector transmission. Similar to other geminiviruses, recombination plays a key role in their evolution. The majority of betasatellites are naturally recombinant (20). There is no sequence homology between betasatellites and their helper viruses except for the stem-loop region (21). Betasatellites encode a single gene, β*C1,* that plays a role in producing symptoms, suppressing gene silencing and movement, but that is dispensable for replication (20, 22, 23). This gene can be removed from the betasatellite to increase the cargo capacity for gene/DNA delivery into plants. Furthermore, betasatellites contain an A-rich region and a satellite-conserved region (SCR). The SCR is approximately 200 nt in length and includes a predicted hairpin structure, which is similar to the origin of replication for the geminiviruses (24). The A-rich region is characterized by a high adenosine content ranging from 57 to 65% and is typically 160 to 280 nt in size (24). It is thought that this region plays a role in the stability of betasatellite molecules by increasing their size, thereby enabling efficient encapsidation and systemic movement (25). Furthermore, experimental transreplication of cotton leaf curl Multan betasatellite (CLCuMB) has been observed in the presence of several monopartite geminiviruses including BCTV-Svr (formerly BSCTV) (26). Co-infection of the betasatellite with the helper virus (e.g., BCTV) in the same cell makes this satellite molecule a suitable candidate for studying homologous recombination with their helper viruses (27).

Geminiviral replicons (GVRs), which contain genes essential for virus replication, have been used to improve homologous DNA recombination (HDR) when delivering CRISPR-Cas components in tobacco, tomato, potato, and rice (28–31). Similarly, the beet curly top virus replicon has been used to efficiently deliver CRISPR/Cas12a components, thereby improving the rate of mutagenesis and HDR in *Nicotiana benthamiana* plants (32). However, the detailed effects of GVRs (e.g., Rep and other viral genes) on the HDR pathway in plant cells are unknown. Therefore, understanding the role of GVRs, including the BCTV replicon, may shed light on both viral homologous recombination and HDR in plants. This could help to optimize the use of GVRs to improve HDR via the CRISPR/Cas system in plants in the future. In addition, recombination plays a significant role in the evolution, diversity, and emergence of new pathogenic species in the *Geminiviridae* family.

There is evidence that viral genome recombination plays a critical role in driving host switching and the emergence of geminiviruses, which makes them such successful plant pathogens (33). For example, curtoviruses possess chimeric genomes that include sequences derived from other BCTV strains or other viral species (34), and thus several new strains or species associated with curly top disease in sugar beets and other crops have been identified. These new BCTV variants are likely the result of recombination during mixed infections and selection pressure (35). However, the processes by which recombination occurs within ssDNA viruses have been poorly characterized, and the role of geminiviral genes in DNA recombination is unknown (36). Therefore, a combination of betasatellite and helper viruses can be used to understand the role of viral genes in homologous recombination in geminiviruses.

Various approaches have been used to evaluate the evolutionary and pathological consequences of naturally occurring recombinants between geminiviruses (27). These include the co-inoculation of closely related geminiviruses (37), the co-infection of heterodimeric infectious constructs, a mixture of two reciprocal chimeras, or inoculation of near-lethal artificially generated recombinants (27). However, a model that provides a simple and quantitative approach to assess the homologous recombination frequency (HRF) between geminiviruses and allows the study of the role of viral genes in the HRF is lacking. In this study, we developed a visual and quantitative model to test the HRF between BCTV and betasatellite replicons in locally co-infected plant tissues. Using this model, we investigated the role of BCTV complementary-sense genes (*C1, C2, C3,* and *C4*) and structural elements (e.g., iteron and stem-loop) in HR between BCTV and CLCuMB replicons.

## MATERIALS AND METHODS

### Construction of a CLCuMB replicon from a full-length clone

**Fig 1:**
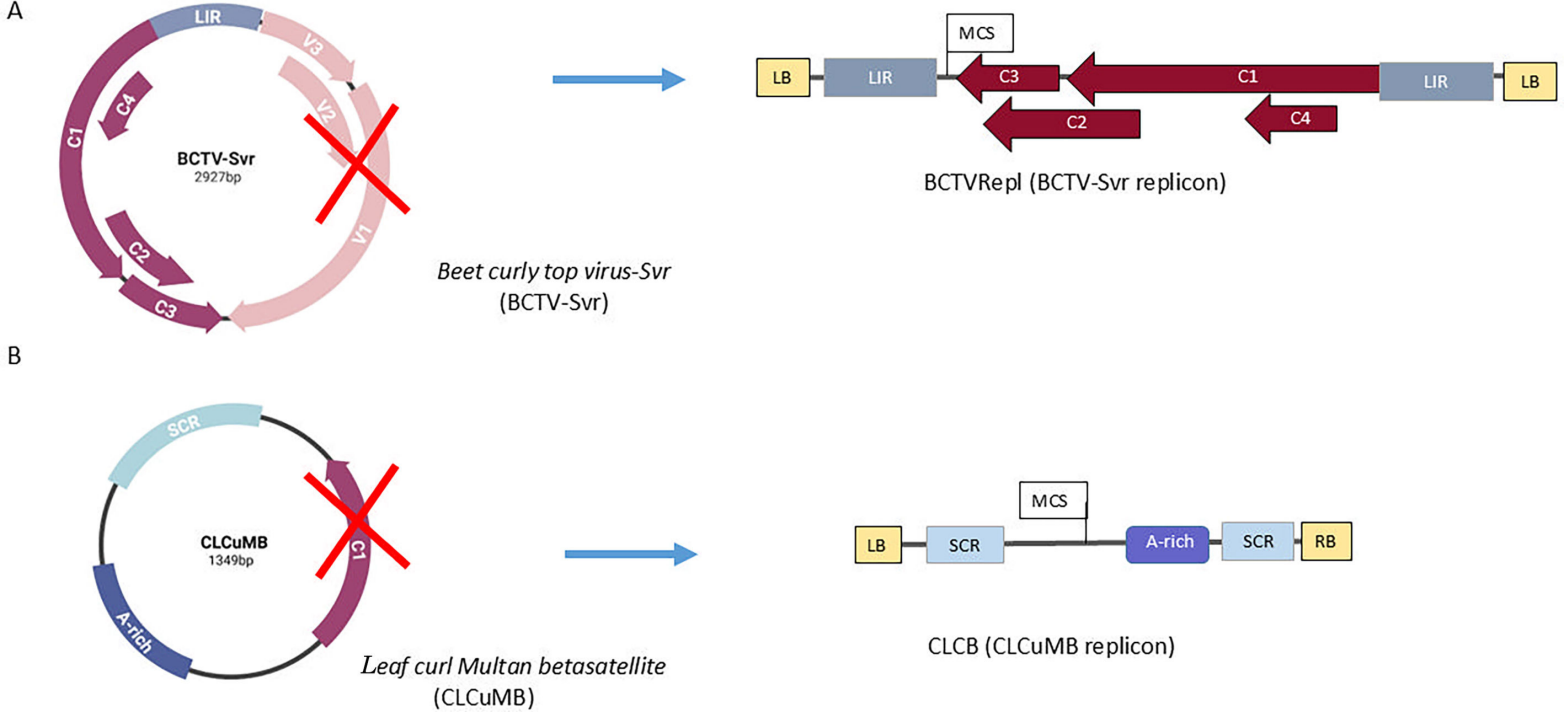
Genomic maps and replicon construction for BCTV-Svr and CLCuMB. (**A**) Genomic maps for BCTV-Svr full-length and the derived BCTV replicon (BCTVRepl), showing the viral genome organization (genes and long intergenic region), the introduced multiple cloning site in the BCTVRepl, which lacks virion genes (*V1*, *V2*, and *V3*). BCTV-Svr indicates the severe strain of BCTV (formerly known as BSCTV). (**B**) Genomic maps for the full-length CLCuMB and the derived CLCB replicon (CLCB), showing the satellite genome organization including the A-rich SCR and β*C1* gene. The position of the introduced multiple cloning site is indicated. Both replicons were subcloned into a binary vector. The left border (LB) and right border (RB) are shown.

A DNA fragment (size 308 nt) of CLCuMB DNA (GenBank accession no. AJ298903) containing the SCR was amplified by PCR using pzf-CLCB-F/CLCMB-SpeI-R primers (Table S1). Another DNA fragment (993 nt in size) containing the satellite A-rich region and the SCR was amplified by PCR using CLCB-SpeI-F/pzf-CLCB-R primers (Table S1). These fragments were subcloned into the *Nco*I/*Sal*I site of a binary vector, pZFN, using the Gibson assembly method (38) to produce the pZFN-CLCBRepl construct. A multiple cloning site (*Asc*I, *Xma*I, *Sma*I, and *Sca*I) was introduced into the *Spe*I site of pZFN-CLCBRepl (hereafter referred to as CLCB) to facilitate downstream subcloning. The map of the virus and betasatellite was generated using the Biorender tool (https://www.biorender.com/) (Fig. 1). This construct was transferred into *Agrobacterium* cells (strain C58) by electroporation (Multiporator, Eppendorf).

### Local co-infiltration and qPCR assay

Sugar beet (*Beta vulgaris*) and *N. benthamiana* plants were grown in a glasshouse at 25°C under a 16 h light/8 h dark photoperiod. The BCTV replicon (hereafter referred to as BCTVRepl) was prepared by replacing the virion sense genes (*V1, V2,* and *V3*) of BCTV-Svr with a multiple cloning site and then subcloning into the pZFN binary vector (32). This construct was transformed into *Agrobacterium* cells (strain C58). *Agrobacterium* cells containing BCTVRepl and CLCB were grown in Luria-Bertani (LB) media at 28°C to reach an OD_600_ = 1. For the local infiltration assay, *Agrobacterium* cells were precipitated and then resuspended in MES buffer (10 mM MES, 10 mM MgCl_2_, 100 µM acetosyringone) to reach an OD_600_ = 0.5. After 3 h in the dark, cells were co-infiltrated into the cotyledons of *Beta vulgaris* seedlings and *N. benthamiana* leaves. As a negative control, tissues were co-inoculated with CLCB cells and *Agrobacterium* cells containing empty binary vector (pZFN) to reach the same OD_600_ = 0.5.

Locally infiltrated tissues were collected after 1 and 6 days (three biological replicates including a pool of three leaf sections). Total DNA was extracted using the cetyltrimethylammonium bromide (CTAB) method (39). After *Dpn*I restriction enzyme digestion (Thermo Fisher, USA), the DNA concentration was measured using Nanodrop ND-1000 (Thermo Fisher Scientific; Denovix, Wilmington, USA) and diluted with sterile water to a final concentration of approximately 100 ng/µL.

For performing the quantitative polymerase chain reaction (qPCR), 100 ng of extracted DNA was used in a 15 µL volume reaction containing 1× iTaq Universal SYBR Supermix (Bio-Rad), 0.450 µM of each primer. For quantification of BCTVRepl and CLCB, BCTV-F3/BCTV-R3 and CLCB-F/CLCB-R primers (Table S1) were used, respectively. The qPCR was performed on a CFX96 Real-Time System C1000 Touch Thermal Cycler (Bio-Rad, USA). Reaction conditions were set as described (40). As an endogenous control, F F-box (accession number Niben.v0.3.Ctg24993647) (41) was used as a reference to normalize the viral and satellite DNA accumulation in infected *N. benthamiana* plants. Three biological replicates were tested for each virus and statistically analyzed using R software (Tukey’s test, *P* < 0.05).

### Construction of reporter plasmids and microscopic assays

**Fig 2:**
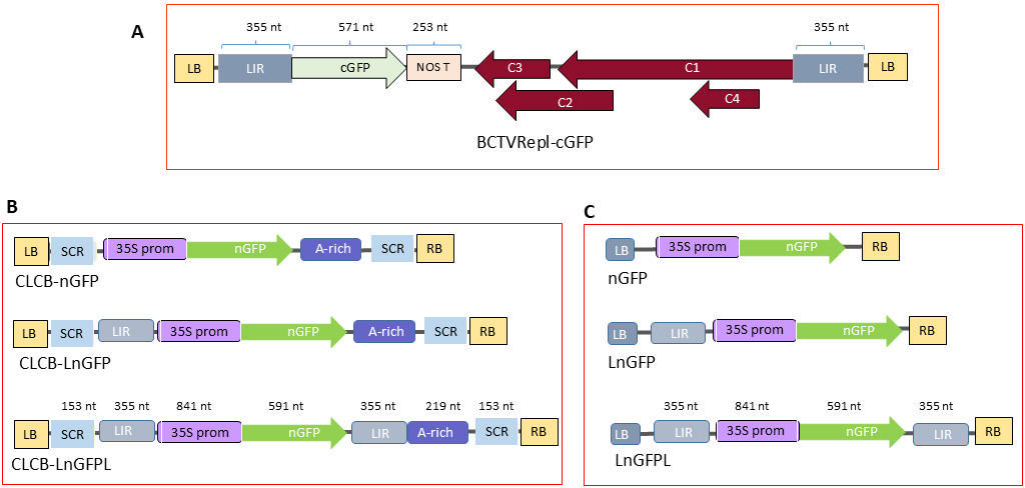
Schematic maps of BCTVRepl, CLCB, and T-DNA constructs. (**A**) Genomic maps for BCTVRepl-cGFP show the viral genome organization including *C1*, *C2*, *C3*, and *C4* genes, two intergenic regions (LIR), and the introduced C-terminus of the GFP insert (cGFP). The size of the features is indicated in nt. (**B**) Genomic maps for CLCB replicon constructs containing the SCR and A-rich region and additional N-terminus of GFP (CLCB-nGFP), N-terminus plus LIR (CLCB-LnGFP), or N-terminus plus two LIR sequences (CLCB-LnGFPL). (**C**) T-DNA constructs encoding the N-terminus of GFP (nGFP), N-terminus plus IR (LnGFP), or N-terminus plus two LIR sequences (LnGFPL).

The C-terminus of the 35S-GFP cassette (931 nt in size) was amplified by PCR using GFP-Spe-F/Nos-Spe-R primers and subcloned into the *Spe*I site of BCTVRepl (32) to produce BCTVRepl-cGFP (Fig. 2).

The N-terminus of the 35S-GFP cassette plus LIR of BCTV (1869 nt in size) was amplified by PCR using BS-LIR-Sma-F/GFP-Hind-R primers and BCTVRepl-GFP (32) as DNA template. These DNA fragments were subcloned into *Sma*I/*Hind*III sites in CLCB and pZFN binary vectors to produce CLCB-LnGF and pZFN-LnGF (referred to as LnGFP), respectively. To produce pZFN-nGFP (called nGFP), the LIR fragment (355 nt in size) was removed from pZFN-LnGF by *Asc*I digestion and religation. This enzyme cuts both sides of the LIR and releases this fragment. Furthermore, the LIR fragment of BCTV was amplified by PCR using BS-LIR-Hind-F/BS-LIR-Hind-FR primers and then subcloned into the *Hind*III site of pZFN-LnGFP and CLCB-LnGFP to produce pZFN-LnGFPL (referred to as LnGFPL) and CLCB-LnGFPL constructs. Schematic maps of these constructs were generated using the Biorender tool (https://www.biorender.com/).

*Agrobacterium* cells containing BCTVRepl-cGFP, T-DNA (nGFP, LnGFP or LnGFPL), CLCB constructs (CLCB-nGFP, CLCB-LnGFP, or CLCB-LnGFPL), and control (35S-GFP) were grown in LB media at 28°C to reach an OD_600_ = 1. For the local infiltration assay, *Agrobacterium* cells were first precipitated and then resuspended in MES buffer to reach an OD_600_ = 1, and after 3 h in the dark, cells containing BCTVRepl-cGFP were co-infiltrated with either T-DNA or CLCB cells into *N. benthamiana* leaves. For negative control, *N. benthamiana* leaves were co-inoculated with cells containing only CLCB-nGFP or BCTVRepl-cGFP constructs together with *Agrobacterium* cells containing empty binary vector (pZFN) to reach the same OD_600_ = 0.5.

To visualize GFP in the infiltrated tissues, GFP signals (488 nm excitation and 490–514 nm emission) were captured by confocal laser scanning microscopy (Carl Zeiss, LSM710, Jena, Germany).

To quantify DNA copies for N-terminus GFP in T-DNA or CLCB constructs by qPCR, 35S-s/GFP-R4 primers that target only the N-terminus of GFP but not the overlapping (homologous) sequence were used. For this assay, a pool of three leaf sections from the infiltrated patches was collected for each biological replicate to minimize variation between the biological replicates. Total DNA was extracted from the co-infiltrated patches with BCTVRepl-cGFP plus either T-DNA (nGFP, LnGFP, or LnGFPL) or CLCB (CLCB-nGFP, CLCB-LnGFP, or CLCB-LnGFPL) constructs at 3 dpi, digested with *Dpn*I restriction enzyme, and used in the qPCR assay as described above.

### Isolation of protoplasts and quantification of GFP cells

Leaf tissues were collected from the infiltrated patches after 3 days and used for protoplast isolation based on the method described in reference 41 with some modifications (Fig. S1).

The infiltrated leaf tissue (approximately 200 mg) was cut into small pieces (2–5 mm) with a scalpel and incubated with 2.5 mL of the enzyme solution containing half macrosalts of SH medium (200 mg/L MgSO_4_·7H_2_O, 100 mg/L CaCl_2_·2H_2_O and 150 mg/L NH_4_H_2_PO_4_), 0.4 M sorbitol, 4 mM CaCl_2_, 12.5 mM MES-KOH (pH 5.7), 1% cellulase Onozuka R10 (Duchefa-Biochemie, Haarlem, Netherlands), 0.15% pectinase Macerozyme R10 (Duchefa-Biochemie, Haarlem, Netherlands), and 0.4% hemicellulose driselase (Sigma-Aldrich, Germany). Enzyme solutions (10×) were prepared, clarified by centrifugation, and stored at −20°C. Protoplasts were isolated at 22°C with gentle shaking (50 rpm) for 16 h. The protoplast suspension was filtered through a nylon mesh (40 µm pore size), transferred to 2 mL tubes, and centrifuged at 100 *× g* for 5 min at room temperature. The pellet of protoplasts was suspended in 2 mL of 0.5 M sorbitol containing 2.5 mM CaCl_2_ (Sorb-Ca), incubated for 5 min at room temperature, and centrifuged at 100 *× g* for 5 min at room temperature (the procedure was repeated twice). The sedimented protoplasts were suspended in 0.5 mL of Sorb-Ca and monitored for GFP expression under UV light using an epifluorescence microscope (Carl Zeiss, AXIO, Jena, Germany) with GFP filter (488 nm excitation and 490–514 nm emission).

Three biological repeats were tested for each treatment. For each sample, five sections were randomly imaged under UV and normal light, and the ratio of GFP-expressing cells (hereafter referred to as GFP cells) to the total number of cells was recorded and statistically analyzed using R software (Tukey’s test, *P* < 0.05). To control the efficacy of each infiltration assay, the ratio of GFP cells was measured in the 35S-GFP infiltrated control tissues. The GFP ratio of each treatment was then normalized to the 35S-GFP control.

### Mutation of complementary-sense genes in the BCTV replicon

A fragment (size 1,536 nt) of the BCTV replicon was removed from BCTVRepl-cGFP after *Kpn*I digestion and religation to produce BCTVRepl-cGFP-d which lacks the complementary-sense genes.

To introduce a premature stop codon into the *C2* gene of the BCTV replicon without affecting the overlapping gene, *C1*, two overlapping DNA fragments were amplified by PCR using BC-F4/BSC-C2m-R1 and BSC-C2m-F/BS-R2 primers and BCTVRepl-GFP as DNA template. These DNA fragments (size 698 and 1,247 nt) were gel extracted and used as DNA templates to amplify cGFP C2m with introduced mutations (tga>taa) at the N-terminus of the *C2* gene using BC-F4/BS-R2 primers. This fragment (size 1,925 nt) was subcloned into the *Kpn*I/*Pme*I site of the BCTVRepl-cGFP-d vector to produce the BCTVRepl-cGFP *C2m* construct. A similar cloning approach was used to introduce a premature stop codon in C3, C4, and C2/C3/C4 genes to produce BCTVRepl-cGFP C3m (ttg>tag), BCTVRepl-cGFP C4m (tta>taa and tcg>tag), and BCTVRepl-cGFP C2m/C3m/C4m constructs. All constructs were individually transferred into *Agrobacterium* cells (strain C58) by electroporation.

*N. benthamiana* leaves were co-infiltrated with CLCB-nGFP and each of the BCTVRepl-cGFP mutant constructs (C2m, C3m, C4m, and C2m/C3m/C4m). As a control, the wild-type BCTVRepl-cGFP construct was co-infiltrated. After 3 days, protoplast cells were prepared, and the ratio of GFP cells was analyzed as described above. The integrity and stability of the introduced mutants were tested in the co-infiltrated tissue by PCR and sequencing for each mutant construct.

A subsample (three biological replicates including a pool of three leaf sections) was collected for DNA preparation, *Dpn*I restriction digestion, and then quantification of BCTVRepl-cGFP mutants (C2m, C3m, C4m, and C2m/C3m/C4m) in each treatment by qPCR as described above. For control, tissues co-infiltrated with CLCB-nGFP and wild-type BCTVRepl-cGFP were tested. In addition, this qPCR assay was performed for the same mutant constructs in sugar beet. As an endogenous control, the glyceraldehyde-3-phosphate dehydrogenase gene (KJ784472) (42) was used as a reference to normalize the viral and satellite DNA accumulation in infected sugar beet plants.

### Rolling circle amplification (RCA) assay

Total DNA was extracted from leaf tissues co-infiltrated with BCTVRepl-cGFP and LnGFP or LnGFPL using the CTAB method (39). As a control, DNA was extracted from leaf tissues infiltrated with BCTVRepl-cGFP only.

Circular DNA was amplified with phi29 polymerase using the TempliPhi Kit (Merck, Darmstadt, Germany) according to the manufacturer’s protocol. Approximately 20 ng of total nucleic acids was dissolved in 5 µL sample buffer, denatured at 95°C for 3 min, and cooled down to room temperature. After adding 5 µL reaction buffer and 0.2 µL enzyme mix, the reaction was incubated for 18 h at 30°C and stopped at 65°C for 10 min.

Aliquots of nucleic acids (500 ng) in a volume of 15 µL were digested with FastDigest *Sca*I restriction enzyme (Thermo Fisher Scientific, USA) for 20 min according to supplier’s recommendation. The restriction products were run on a 1% agarose gel and stained with GelRed (Avanator, USA) after electrophoresis. For control, DNA from BCTVRepl-cGFP infected tissues was digested with FastDigest *BamH*I restriction enzyme and separated on the same agarose gel.

### Introduction of the mutation into the intergenic region

Two overlapping DNA fragments were amplified by PCR using pZF-F/BSLIRm-R and BSLIRm-F1/pZF-R primers and LnGFP as DNA template. These DNA fragments (size 116 and 1,957 nt) were gel extracted and used as templates to amplify Ln-GFP with introduced mutations (ggtg>ccgc and ggtg>aaaa) on the direct repeat sequences in the LIR. This fragment (size 2,035 nt) was subcloned into the *Sma*I/*Hind*III site of the pZFN binary vector to produce the LnGFP mut-l construct. A similar approach was used to introduce mutations into the stem-loop sequence (ggggccatcc>cacataggaa and ggatggcccc>tagccttact) and additionally delete 9 nt (taatattac) from the loop sequence in the LnGFP construct to produce LnGFP mut-s, LnGFP mut-s1, and LnGFP mut-d constructs, respectively. All constructs were transformed into *Agrobacterium* cells (strain C58) by electroporation.

*N. benthamiana* leaves were co-infiltrated with BCTVRepl-cGFP and each mutant construct (LnGFP mut-l, LnGFP mut-s or LnGFP mut-d). The wild-type LnGFP construct was co-infiltrated as a control. After 3 days, protoplast cells were prepared, and the ratio of GFP cells was analyzed as described above.

### Nanopore Cas-targeted sequencing (nCATS)

Total DNA was extracted from leaves infiltrated with CLCB-nGFP and BCTVRepl-cGFP. Leaf tissues (500 mg) were ground to powder under liquid nitrogen, and DNA was then extracted using the DNeasy plant kit (Qiagen). DNA was repaired using NEBNext FFPE DNA Repair Mix (NEB), and then small fragments were removed using Short Read Eliminator reagent (Circulomics). Samples were quantified by fluorescence (Qubit dsDNA HS assay, Thermo Fisher).

The nCATS assay was performed as described (43) with some modifications. For the preparation of Cas12a ribonucleoproteins, Alt-R CRISPR-Cas12a crRNAs targeting the N- and C-terminus of GFP (Fig. S2) were synthesized by IDT (Integrated DNA Technologies, Belgium; Table S2). Sequencing was performed for 72 h on a PromethION sequencing instrument (ONT). Data were basecalled with Guppy v.6.4.2 using the SUP accuracy model.

Sequenced reads were tested for quality and then mapped to the reference GFP sequence using CLC genomics (Qiagen; version 22) with default parameters, 50% coverage, and 80% similarity. For screening the full-length GFP reads, reads (445–9,000 nt in size) were aligned to the reference GFP sequence (720 nt in size) with 100% coverage and 90% similarity.

## RESULTS

### CLCuMB replicon accumulates in BCTVRepl co-infected tissues

The CLCuMB replicon (referred to as CLCB) was constructed by subcloning two DNA fragments of CLCuMB into a binary vector, aiming to remove the β*C1* gene and introduce a multiple cloning site. CLCB contains SCR and A-rich regions but lacks the β*C1* gene (Fig. 1). The qPCR data showed that CLCB accumulated at a high level in the locally co-infiltrated tissues in the presence of BCTVRepl after 6 days (Fig. S3). The control CLCB showed the same level of DNA accumulation between days 1 and 6 in the absence of BCTVRepl (data not shown). Therefore, these clones were used for downstream gene delivery and HDR assays.

### Developing a model for virus recombination assay

To develop a model for geminivirus recombination assay, a combination of BCTVRepl and CLCB replicons was tested to generate a visible marker gene following homologous recombination between BCTV replicon and CLCB. The C-terminal coding part of the 35S-GFP cassette, which contains 571 nt of GFP coding region and nopaline synthase (NOS) terminator, was subcloned into BCTVRepl to produce BCTVRepl-cGFP (Fig. 2).

Schematic maps for GFP recombination assay (Fig. S4) show nGFP, containing the 35S promoter and the N-terminal of GFP sequence (591 nt in size) and cGFP which contains the C-terminal region of GFP sequence (571 nt in size) and NOS terminator. The common overlap sequence between cGFP and nGFP was 442 nt in size. After recombination between nGFP and cGFP constructs, a complete GFP sequence with visible phenotype appears (Fig. S4). The nGFP was subcloned into CLCB to produce CLCB-nGFP. For comparison, the same nGFP fragment was subcloned into a binary vector and named nGFP.

**Fig 3:**
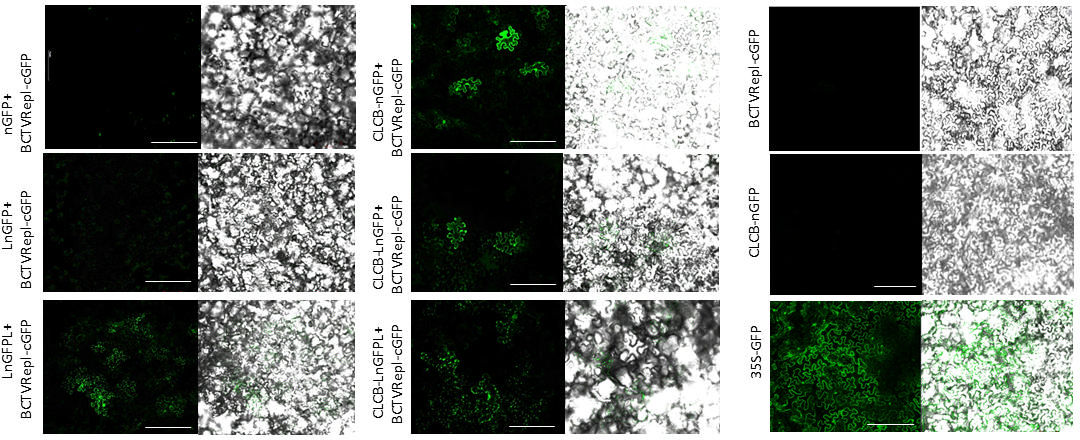
Visualization of homologous DNA recombination using microscopic imaging. Epifluorescence of leaf tissue co-infiltrated with BCTVRepl-cGFP and either CLCB or T-DNA constructs after excitation with 488 nm laser light for GFP using confocal laser scanning microscopy. In the left panel, leaf tissue was co-infiltrated with BCTVRepl-cGFP and a T-DNA construct (nGFP, LnGFP, or LnGFPL); in the middle panel, leaf tissue was co-infiltrated with BCTVRepl-cGFP and a CLCB construct (CLCB-nGFP, CLCB-LnGFP, or CLCB-LnGFPL). Images were taken 3 days after agroinfiltration of *N. benthamiana* leaves. For control, leaves were agro-infiltrated with CLCB-nGFP or 35S-GFP construct. Scale bar = 100 µm.

To further understand the role of LIR in DNA recombination, the LIR sequence of BCTV was introduced into one side or both sides of nGFP or CLCB-nGFP constructs to produce LnGFP, LnGFPL, or CLCB-LnGFP and CLCB-LnGFPL (Fig. 2). Co-infiltration of each construct with BCTVRepl-cGFP in *N. benthamiana* showed GFP expression in the epidermal cells after 3 days (Fig. 3). However, in plants co-infiltrated with only nGFP construct, no GFP cells were observed using confocal laser scanning microscopy (CLSM) and protoplast assay. Leaf tissue co-infiltrated with LnGFP or LnGFPL produced a number of GFP cells. In contrast, a much higher number of cells with GFP fluorescence was observed in all leaves co-infiltrated with CLCB-nGFP, CLCB-LnGFP, or CLCB-LnGFPL (Fig. 3). The control plants that were infiltrated with the single construct, CLCB-nGFP or BCTVRepl-cGFP, did not produce GFP-fluorescent cells. This experiment was repeated at least three times with the same results.

### High recombination rate between betasatellite and BCTV replicon

**Fig 4:**
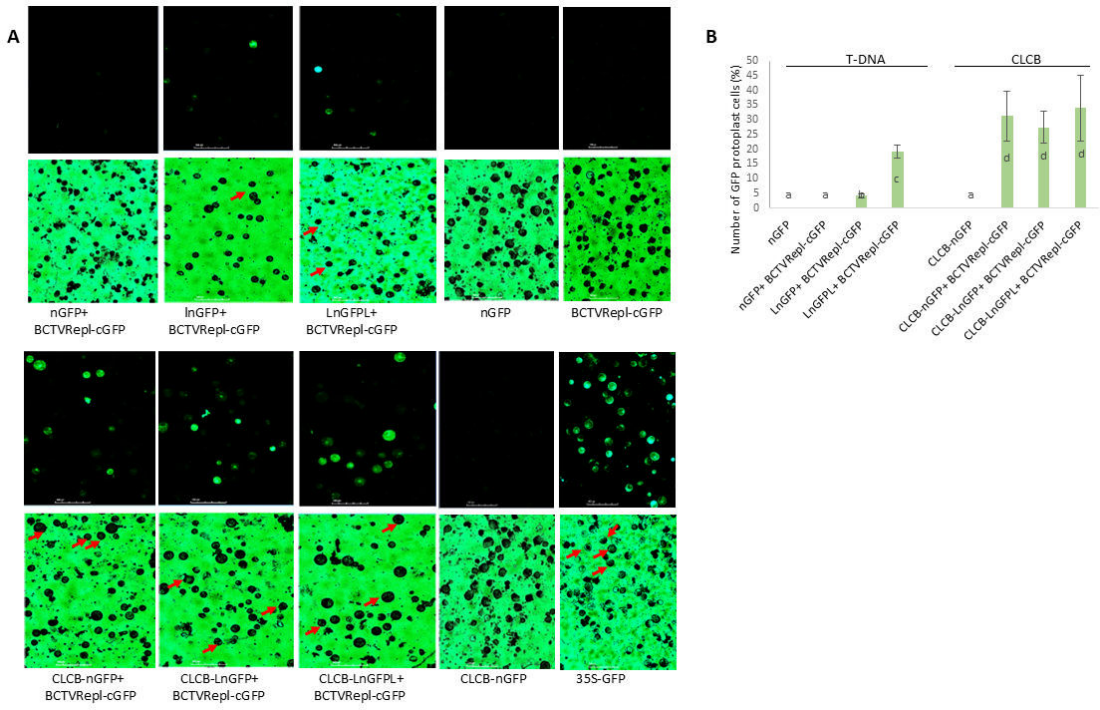
Comparison of homologous DNA recombination rate by quantification of GFP protoplast cells. (**A**) GFP-fluorescent cells were compared in leaf tissue co-infiltrated with BCTVRepl-cGFP and either T-DNA (nGFP, LnGFP, or LnGFPL) or CLCB (CLCB-nGFP, CLCB-LnGFP, or LnGFPL) constructs. Red arrows indicate representative cells that produced green fluorescence. For each treatment, a representative UV (top panel) and light (bottom panel) microscopy image is shown. Scale bars represent 100 µm. (**B**) Analysis of the ratio of GFP cells in co-infiltrated tissues. For each treatment, three biological replicates and five images under UV and normal light were analyzed. Error bars indicate standard deviations. Bars with the same letter indicate statistically insignificant differences (*P* < 0.01).

To quantify and compare the GFP recombinant cells, protoplasts were prepared from the infiltrated patches, and the ratio of GFP cells to total cells was calculated. GFP cells were not detected in leaf patches co-infiltrated with nGFP and BCTVRepl-cGFP, but plants co-infiltrated with LnGFP produced GFP cells (4% on average), and the number of GFP cells was significantly higher (19%) in tissues co-infiltrated with LnGFPL (Fig. 4). In the 35S-GFP control, 96.3% (±2.4) of the cells showed fluorescence, reflecting the high efficiency of the infiltration assay.

A higher rate of GFP cells (27% to 34%) was observed in tissues co-infiltrated with CLCB-nGFP, CLCB-LnGFP, or CLCB-LnGFPL compared with T-DNA constructs (Fig. 4), while single infiltration of each construct did not produce fluorescent cells (data not shown). This indicates that both the N-terminus (149 nt) and the C-terminus (129 nt) of the GFP coding region are required for the production of green fluorescence in cells. There was no significant difference between CLCB-nGFP, CLCB-LnGFP, and CLCB-LnGFPL constructs for recombination with BCTVRepl-cGFP (Fig. 4).

### Viral DNA accumulation affects DNA recombination frequency

To test the effect of DNA accumulation on the number of GFP-fluorescent cells, the accumulation of N-terminal GFP coding region in tissues co-infiltrated with nGFP, LnGFP, or LnGFPL together with BCTVRepl-cGFP was measured using qPCR. To minimize residual inoculum DNA in the replication assays, the extracted DNA was digested with DpnI restriction enzyme before the qPCR assays. There was no significant difference in the accumulation of N-terminal DNA in tissues co-infiltrated with nGFP and LnGFP. However, a higher level of N-terminal DNA was accumulated in LnGFPL samples compared with nGFP and LnGFP constructs (Fig. S5). BCTVRepl-cGFP accumulated at a similar and high level (average of 7,680 ± 246) in all treatments.

**Fig 5:**
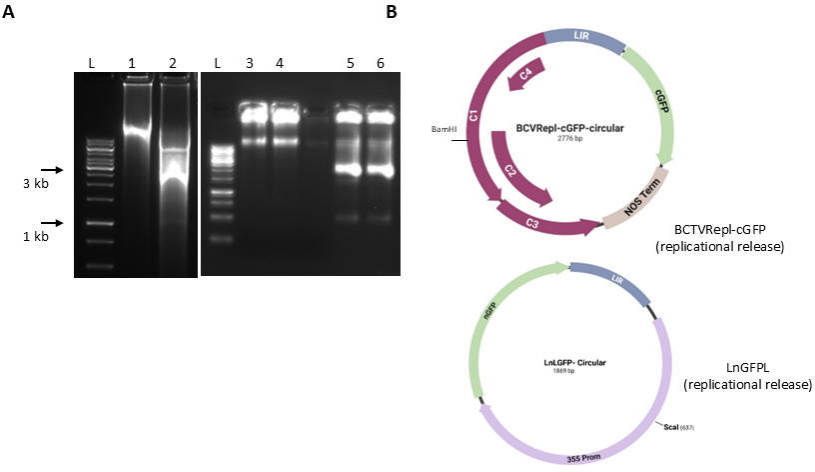
Gel electrophoresis patterns and genetic maps for RCA products at 3 dpi. (**A**) RCA products from leaf tissue co-infiltrated with BCTVRepl and either LnGFP (lanes 3 and 4) or LnGFPL (lanes 5 and 6). The RCA products were digested with a single cutter (*Sca*I restriction enzyme) to release the amplified DNA in lanes 5 and 6. RCA products from tissues infiltrated with only BCTVRepl-cGFP were digested with *BamH*I (lane 2), releasing the replicated form of BCTVRepl with an approximate size of 2,776 nt. Lane 1 represents the undigested control for BCTVRepl. A total of 1 kb DNA ladder was used on the left side of the gel. (**B**) Schematic maps of released circular DNA in leaf tissues co-infiltrated with BCTVRepl-cGFP and LnGFPL. The position of the restriction enzyme is indicated for each DNA.

The presence of two LIRs in LnGFPL may lead to homologous DNA recombination and the production of a new replicon containing nGFP that can replicate in the presence of the helper virus. To test the replicational release of a new replicon from LnGFPL constructs in the presence of helper BCTVRepl-cGFP, total DNA from tissues co-infiltrated with LnGFPL and BCTVRepl-cGFP was used in the RCA assay. As a control, tissues infiltrated with BCTVRepl-cGFP alone or in combination with LnGFP were also tested. Digesting the RCA product with the *Sca*I restriction enzyme released DNA fragments (approximately 1,800 nt in size) in tissue co-infiltrated with LnGFPL, but not in tissue co-infiltrated with LnGFP (see Fig. 5). The *Sca*I restriction enzyme only cleaves the insert fragments in the LnGFPL and LnGFP constructs, not the plasmid backbone. This was to avoid the possibility of the delivered plasmid vector being detected and amplified by RCA. The control BCTVRepl-cGFP sample produced the expected size of 2,776 nt after digestion with the *BamH*I enzyme, which cleaves the released replicon at a single site.

More N-terminal DNA was accumulated in CLCB-LnGFPL compared to CLCB-LnGFP and CLCB-nGFP (Fig. S5). In addition, the accumulation of CLCB was tested by qPCR. A striking level of CLCB DNA was accumulated in tissues co-infiltrated with CLCB-LnGFPL compared to CLCB-LnGFP and CLCB-nGFP. The level of DNA in CLCB-LnGFP was 1.5 times higher than that in CLCB-nGFP (Fig. S5).

### Genetic requirements for homologous recombination by BCTV replicon

**Fig 6:**
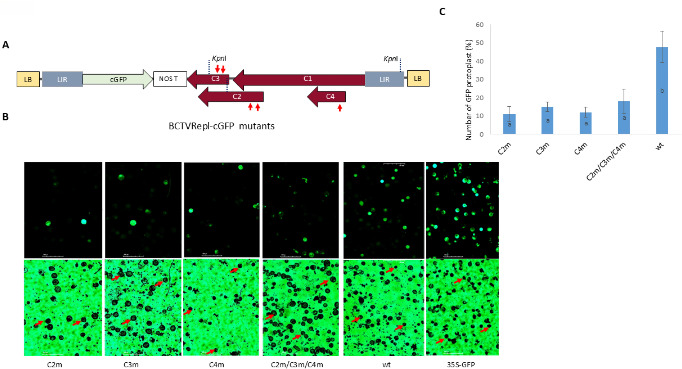
Effect of mutation of BCTVRepl-cGFP genes (C1, C2, C3, and C4) on recombination between BCTVRepl-cGFP and CLCB-nGFP. (**A**) Schematic maps for BCTVRepl-cGFP mutants show the viral genome organization and the position of premature stop codons in each gene as indicated by red arrows. The *Kpn*I restriction site was used to construct BCTVRepl-cGFP mutants. (**B**) GFP-fluorescent cells were compared in leaf tissues co-infiltrated with CLCB-nGFP and each of the BCTVRepl-cGFP mutants (C2m, C3m, C4m, or C2m/C3m/C4m). For control, intact BCTVRepl-cGFP (wt) was co-infiltrated with CLCB-nGFP. For each treatment, a representative UV (top panel) and light microscopy (bottom panel) image is shown. Red arrows indicate representative cells showing green fluorescence. Scale bars represent 100 µm. (**C**) For each treatment, three biological replicates and five sections were imaged under UV and normal light and statistically analyzed. Error bars indicate standard deviations. Bars with the same letter indicate statistically insignificant differences (*P* < 0.01).

To test the role of the BCTV replicon genes (*C1, C2, C3,* and *C4*) in viral recombination in the developed model system, mutants of BCTVRepl-cGFP with one or two premature stop codons in each gene (*C2, C3,* and *C4*) were prepared (Fig. 6). In addition, a BCTVRepl-cGFP construct with stop codons in all three genes (*C2, C3,* and *C4*) was prepared. This construct was expected to encode only *C1*.

CLCB-nGFP and each of the BCTVRepl-cGFP mutant constructs (*C2m, C3m, C4m* or *C2m/C3m/C4m*) were co-infiltrated into *N. benthamiana* leaves. As a control, the wild-type BCTVRepl-cGFP construct was co-infiltrated. After 3 days, protoplast cells were prepared, and the ratio of GFP cells was analyzed as described above. The number of GFP-fluorescent cells was drastically reduced to about 10% of total protoplasts in all BCTVRepl-cGFP mutants compared with wild-type BCTVRepl-cGFP (about 50% of total protoplasts) (Fig. 6). The high rate of GFP-fluorescent cells (90% ± 5.7) in the control 35S-GFP indicated the high efficacy of this infiltration assay.

To test the effect of these mutations on the replication of BCTVRpl-cGFP, total DNA was extracted from *N. benthamiana* tissues co-infiltrated with BCTVRepl-cGFP mutants (*C2m, C3m, C4m* or *C2m/C3m/C4m*), and the accumulation of BCTVRepl-cGFP mutants was tested by qPCR. To further confirm these results, the assay was also performed in sugar beet plants by co-infiltration of these mutants into cotyledons. The level of BCTVRepl-cGFP mutants was reduced to about 20%, 64% and 6% in *C3m, C4m,* and *C2m/C3m/C4m* treatments compared to the wild-type BCTVRepl-cGFP in *N. benthamiana* plants and 4%, 76% and 3% in sugar beet plants, respectively (Fig. S6). However, in the *C2m* treatment, the level of DNA was not changed compared to the wild-type replicon in both *N. benthamiana* and sugar beet assays.

### *C1* gene and stem-loop sequence have a role in recombination

**Fig 7:**
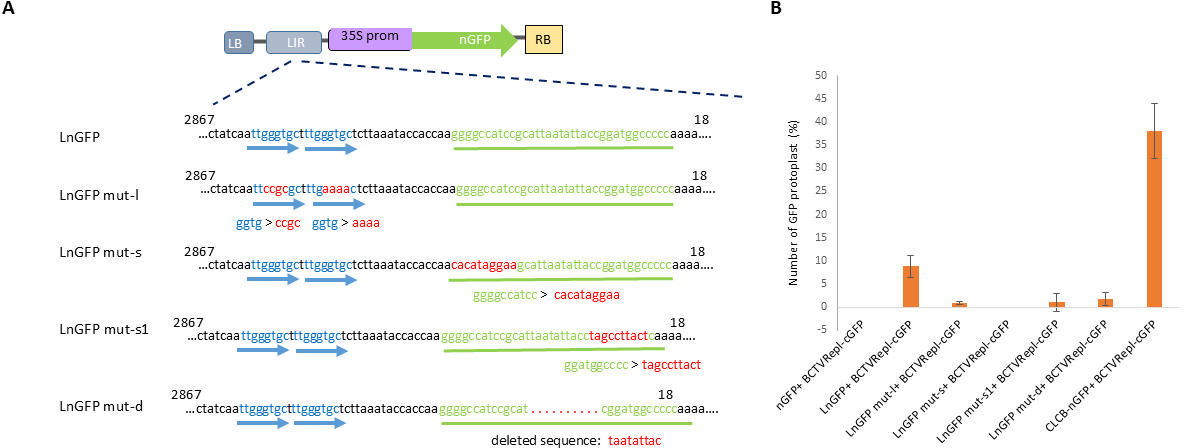
Mutations in the intergenic region (IR) in the LnGFP construct for their effects on recombination with BCTVRepl-cGFP. (**A**) Genomic map of LnGFP and sequences show the position of mutated sequences in the intergenic region (nucleotide position 2867 to 18 according to the BCTV-Svr genome sequence). The LnGFP mut-l construct contains mutations in the direct repeats (indicated by blue arrows). The mutated nucleotides are shown in red letters. The stem-loop sequence is in green color. The mutated sequences in the stem-loop are shown in red in LnGFP mut-s and LnGFP mut-s1. The position and deleted nucleotides in the stem-loop in LnGFP mut-d are shown with red dots. (**B**) Comparison of homologous DNA recombination rate by quantification of GFP protoplasts. GFP-fluorescent cells were compared in leaf tissues co-infiltrated with BCTVRepl-cGFP and each mutant construct (LnGFP mut-l, LnGFP mut-s, LnGFP mut-s1 or LnGFP mut-d). For each treatment, three biological replicates and five sections each were imaged under UV and normal light. The ratio of GFP cells was compared to the wild-type construct, LnGFP. As a control, nGFP or CLCB-nGFP were co-infiltrated with BCTVRepl-cGFP and analyzed in the same experiments. Error bars indicate standard deviations. Bars with the same letter indicate statistically insignificant differences (*P* < 0.01).

To test the role of the *C1* gene in GFP recombination between BCTVRepl-cGFP and LnGFP, the putative *C1* binding sites on the LIR of the LnGFP construct were mutated (cctg>ccgc and ggtg>aaaa) to produce LnGFP mut-l (Fig. 7). This construct was co-infiltrated with BCTVRepl-cGFP into *N. benthamiana* leaves. After 3 days, protoplasts were prepared, and the ratio of GFP-fluorescent cells was analyzed. The number of GFP-fluorescent cells was drastically reduced in tissues co-infiltrated with LnGFP mut-l compared with wild-type LnGFP (Fig. 7).

In addition, the potential role of the stem-loop sequence, which is involved in virus replication and interaction with Rep, was tested by mutating this element in the LnGFP construct. Three LnGFP mutants, including LnGFP mut-s, LnGFP mut-d, and LnGFP mut-s 1, were constructed. Each mutant construct was co-infiltrated with BCTVRepl-cGFP in *N. benthamiana* leaves, and the ratio of GFP-fluorescent cells was analyzed and compared with wild-type LnGFP. LnGFP mut-d, LnGFP mut-s, and LnGFP mut-s1 mutants showed no GFP cells in tissues co-infiltrated with BCTVRepl-cGFP (Fig. 7). The infiltration efficiency in the 35S-GFP control was relatively low (83.6% ± 4.7). Therefore, the data from this assay were normalized to the average ratio of GFP cells (93%) in other experiments for better comparison.

### Targeted nanopore sequencing revealed GFP recombinant sequences

To detect the recombinant GFP sequences, we first used PCR to amplify the new recombinant GFP but produced false-positive data due to template switching by Taq polymerase. Therefore, to overcome this problem, we had to use a non-amplification method such as targeted nanopore sequencing with Cas12a-guided adaptor ligation method (43).

A total of 3,100,165 reads with an average length of 1,633 nt (size from 50 to 9,000 nt) were sequenced in a DNA sample from a leaf co-infiltrated with CLCB-nGFP and BCTVRepl-cGFP. Standard parameters (50% coverage and 80% similarity) were used to map the reads to the target sequences. Analysis of the sequences showed that 19.01% of the reads (589,342 out of 3,100,165) with an average length of 650 nt were mapped to the CLC-nGFP, 53.14% of the reads (1,647,340 out of 3,100,165) with an average length of 1,495 nt were mapped to the BCTVRepl-cGFP genome, and 15.86% of the reads (406,274 out of 3,100,165) with an average length of 1,137 nt were mapped to the *N. benthamiana* genome (https://nbenthamiana.jp/). Only 13.9% of the reads (431,533 out of 3,100,165) with an average length of 697 nt were mapped to the complete GFP coding region.

**Fig 8:**
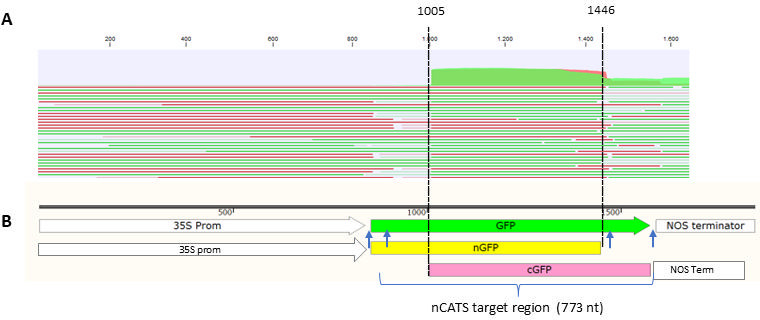
A representative read mapping to the 35S-GFP target sequence. (**A**) nCATS reads in leaf tissues co-infiltrated with BSCTV-cGFP and CLCB-nGFP were mapped to the 35S-GFP sequence using the CLC genomics tool (mapping parameters, 100% coverage and 90% similarity). Red and green lines represent forward and reverse sequence reads, respectively. Most reads (1,647,340 reads) mapped to the overlapping GFP sequence between 1,005 and 1,446 nt, while 10,623 reads cover the recombinant GFP sequences. (**B**) Schematic maps showing the nGFP, cGFP, and nCATS target regions. Small blue arrows indicate four crRNA target regions on either side of the GFP gene in the nCATS assay.

To avoid sequence reads of the N-terminal or C-terminal GFP input DNAs, sequence reads shorter than 442 nt in size were trimmed. Mapping of the longer reads (2,590,612 reads; 443 to 9,000 nt in size) showed that 0.41% (10,623 of 2,590,612 reads) matched the coding region of GFP and covered the recombination point between the C-terminal and N-terminal GFP sequences using more restricted mapping parameters, 100% coverage and 90% similarity (Fig. 8). Because of such restricted mapping parameters to avoid false-positive data, we can expect that some shorter reads were excluded from the actual recombinant DNAs. Therefore, a higher DNA recombination rate than 0.41% can be expected.

Finally, a high level of off-target sequences (15.68%) was recorded in this assay. It has been reported that off-target reads occur more frequently with targeted sequencing such as the Cas system (44). For example, the Cas9 system often has off-target reads as well as sequencing-based targeting of regions (~30% enrichment on target) (43).

## DISCUSSION

Recombination (homologous and non-homologous) is common among geminiviruses and occurs between species and genera. Interspecies recombination has led to significant diversity among geminiviruses, which in several cases has been associated with changes in the host range and pathogenicity (32, 36, 45). In addition, there are examples showing how HDR can be improved by using geminiviral replicons, including the BCTV replicon, to deliver CRISPR/Cas and repair DNA templates efficiently into plant cells (30, 32). However, the potential role of viral genes in HDR has not yet been determined. In this study, we developed an efficient and quantitative model to evaluate the recombination between BCTV and CLCB replicons by generating a visible marker gene. This model was based on the recombination assay in baculoviruses (insect viruses with dsDNA genome), which was applied to investigate the function of viral genes in viral DNA replication and the effectiveness of HDR between the viral genome and plasmid DNAs (46). Using this model, we identified BCTV genes and DNA elements that play a role in HDR between the BCTV replicon and the CLCB constructs. To develop this model, a replicon from CLCuMB was initially prepared to remove the β*C1* gene and provide more cargo capacity in this betasatellite. Figure S3 shows that the produced betasatellite replicon (CLCB) replicates in the presence of the BCTV replicon in the locally co-infiltrated tissues, indicating that the β*C1* gene is not essential in CLCB replication. Supporting this result, full-length BCTV has been shown to trans-replicate CLCuMB in tomato, *N. benthamiana,* and datura plants (26); and β*C1* is dispensable for replication of betasatellites (20, 22, 23). The specificity of the interaction between the Rep protein and the DNA elements of the intergenic region in the process of geminiviral replication is controversial (45, 47). Apart from the nonanucleotide sequence, betasatellites share little sequence homology with their helper viruses (25). The mechanism by which betasatellites can achieve such a versatile interaction remains unclear, which may indicate that the interaction between the helper virus Rep protein and betasatellites is not highly specific.

There is evidence for recombination between helper geminiviruses and their associated satellites in naturally infected plants. This occasionally produces new satellite molecules that retain the intergenic region of the helper virus (48–50). For example, recDNA-Ab17 is a naturally occurring recombinant of approximately the same size as betasatellites (DNA β) that contains sequences from both the helper virus (Ageratum yellow vein virus) and betasatellites. It induces symptoms in co-infected plants (51). In addition, HDR between geminiviral DNA and homologous DNA in transgenic plants has been reported between the African cassava mosaic virus mutant and the coat protein gene with flanked homologous sequence to the virus genome (49). The presence of flanked homologous sequences highlighted the requirement of such homologous sequences for DNA recombination in geminiviruses. In our study, tissues co-infiltrated with BCTVRepl-cGFP and CLCB-nGFP, which contained homologous sequence (442 nt in size), produced green fluorescence. Interaction and recombination between BCTVRepl-cGFP and CLCB-nGFP resulted in a high number of green fluorescent cells (approximately 31%), which is consistent with the frequent natural recombination within and between species, as well as across genera, in geminiviruses (52). However, such green fluorescent cells and recombination were not observed in the control samples co-infiltrated with BCTVRepl-cGFP and nGFP, a control T-DNA construct. In contrast, the LnGFP construct, which contains the BCTV-Svr LIR sequence, improved recombination (approximately 5%) compared to the nGFP construct, which lacks the BCTV-Svr LIR sequence (Fig. 4). This indicates that the presence of a homologous sequence (442 nt in size) in the split GFP gene was insufficient for recombination between BCTVRepl-cGFP and nGFP (T-DNA), and the presence of LIR which contains the Rep binding site (iterons) and stem-loop sequence plays a role in the recombination between BCTVRepl-cGFP and LnGFP (T-DNA with LIR). Supporting this hypothesis, the non-random distribution pattern of several microsatellites in begomovirus genomes, mainly in the intergenic region (common region) containing the stem-loop, has been shown to correlate significantly with the recombination breakpoint positions in the genome (48).

A higher number of GFP cells and DNA recombination was observed in tissues co-infiltrated with BCTVRepl-cGFP and LnGFPL compared to tissues co-infiltrated with BCTVRepl-cGFP and LnGFP. This was directly correlated with the accumulation of N-terminal GFP in these tissues (Fig. S5). This indicates that an increased copy number of homologous DNA in LnGFPL leads to more HDR events. In support of this, a higher copy number of homologous DNA (repair template) enhanced HDR in plant cells (30). The higher copy number of N-terminal GFP in the LnGFPL compared to the LnGFP can be explained by the recombination of homologous DNA in the LnGFPL construct in the presence of the helper virus (BCTVRepl-cGFP). The newly produced replicon from the LnGFPL construct can replicate and produce more copies of N-terminal GFP, which recombine with cGFP to produce a higher number of GFP cells. Indeed, the results of the RCA assay confirmed the release of new replicons in tissues co-infiltrated with BCTVRepl-cGFP and LnGFPL (Fig. 5).

However, CLCB-nGFP (lacking LIR) produces a similar number of GFP-positive cells as CLCB-LnGFP and CLCB-LnGFPL. This suggests that recombination occurs at comparable levels across these three groups. This observation implies that the LIR region may not play a significant role in homologous recombination between CLCBs and BCTV-Repl-cGFP or that there is redundancy between the LIR region and SCR in CLCB-LnGFP and CLCB-LnGFPL for their interaction with the Rep protein of the helper virus, BCTV-Repl-cGFP. Additionally, the DNA accumulation of CLCB-nGFP is similar to that of LnGFPL. However, HDR events of CLCB-nGFP were higher (Fig. 4B). This suggests that SCR or other betasatellite sequence structures may play a more critical role than LIR in facilitating HDR in CLCB constructs.

The high number of GFP cells in tissues co-infiltrated with CLCB-nGFP and CLCB-LnGFP compared to LnGFP may reflect the difference in DNA structure between LnGFP (T-DNA; linear DNA) and CLCB-LnGFP or CLCB-nGFP (satellite DNA), which can replicate and produce more DNA (ssDNA and dsDNA) in the presence of BCTV replicon, thereby increasing the chance of recombination between these molecules. Figure S5 shows that the DNA level of LnGFP is similar to and lower than that of CLCB-nGFP or CLCB-LnGFP in the co-infiltrated tissues. This partially explains the lower recombination rate of LnGFP compared to CLCB-LnGFP. Furthermore, the additional LIR sequence in the LnGFP construct had no effect on DNA levels compared to nGFP. Therefore, it is only the structure of the LIR and the possible interaction of BCTVRepl-cGFP with this sequence that are responsible for recombination and the production of fluorescent cells in tissues co-infiltrated with LnGFP. Indeed, mutating the direct repeat elements in the LIR (LnGFP mut-l construct) significantly reduced the number of GFP cells in co-infiltrated tissues compared to wild-type LnGFP. This may suggest that the interaction between the BCTVRepl-cGFP Rep (*C1* gene) and the LIR sequence in the LnGFP is crucial for recombination between BCTVRepl-cGFP and LnGFP. The direct repeat elements contain strain-specific elements for BCTV replication (53).

The stem-loop structure, which is conserved among geminiviruses, is a known hotspot sequence for recombination (48). It is thought that recombination-dependent replication primarily occurs at the origin of replication within the stem-loop (3). The frequent occurrence of DNA recombinants in the mixed infection of tomato yellow leaf curl virus (a *Begomovirus*) infections showed that various regions of the genome contribute unequally to genetic exchange and recombination. In addition to the intergenic region, it has been suggested that secondary structures at cross-over sites favor the discontinuous DNA replication and the switching of the replication complex between homologous regions of the DNA templates (36). Our results also showed that mutations in the stem-loop (LnGFP mut-s and LnGFP mut-s1) and the deletion of the conserved nonanucleotide within the stem-loop structure in LnGFP mut-d impaired the recombination between these mutants and BCTVRepl-cGFP (Fig. 7). This indicates a possible role for the stem-loop structure, the interaction of Rep with these elements, and nicking within the nonanucleotide motif in the recombination between BCTVRepl-cGFP and LnGFP. Supporting this hypothesis, it was demonstrated that nicking within the homologous DNA molecules stimulates HDR in human cells (21).

Recombination between the BCTV replicon and CLCB was established by the visual detection of GFP fluorescence in the protoplast assays that reflect recombination. To further prove the recombination between BCTV replicon and CLCB by sequence analysis, we used the nCATS assay to avoid DNA amplification steps. nCATS data showed that only 0.41% of reads represented the complete GFP target sequence in tissues co-infiltrated with BCTVRepl-cGFP and CLCB-nGFP. However, in protoplast assays, the recombination rate was approximately 31%. Due to the high copy number of CLCB-nGFP and BCTVRepl-cGFP in the co-infiltrated tissues, 19% and 53.14% of the reads were mapped to these molecules, respectively. This made the detection of the recombinant GFP sequences difficult. In addition, 0.41% of the sequence reads only represent the fully sequenced recombinant GFP DNA, excluding false-negative data such as short reads (<442 nt in size) and longer reads (larger than the GFP coding sequence; >720 nt in size), which may also represent GFP recombination. Nevertheless, the sequencing data confirmed the recombination between BCTVRepl-cGFP and CLCB-nGFP at the DNA level.

Given the role of the *C2* gene in inducing host DNA replication and regulating the cell cycle (54), the potential role of this gene and other complementary-sense genes in DNA recombination was tested in plant tissues co-infiltrated with CLCB-nGFP and each one of the BCTVRepl-cGFP mutants. Figure 6 shows that the number of fluorescent cells was drastically reduced in all BCTVRepl-cGFP mutants. This correlated with DNA accumulation in the *C3m* and *C2m/C3m/C4m* constructs, but not in the *C2m* and *C4m* constructs. This suggests that the *C2* and possibly the *C4* genes may play an independent role in HDR between BCTV and CLCB replicons, possibly by activating host genes in the HDR pathway. In support of this scenario, transcriptome analysis by microarray in transgenic *Arabidopsis* plants expressing BCTV *C2* showed that this gene reactivates the cell cycle to restore DNA replication competence (54). However, there are controversial reports on the role of *C2* in viral DNA accumulation. For example, BCTV *C2* has been reported to promote the replication of BCTV and other geminiviruses (45). In a separate report, the mutation of BCTV *C2* resulted in a modest reduction (1.3- to 1.4-fold) of BCTV accumulation in protoplast cells (55), which is consistent with the findings of this study, demonstrating no effect of the *C2* mutant on BCTVRepl DNA accumulation (Fig. S6). Similarly, the *AC2* (*C2* homolog) of Abutilon mosaic virus has been reported to suppress and control virus replication (56). However, the effect of the *C2* mutant on the replication of wild-type BCTV-Svr needs to be investigated.

It has been shown that BCTV-Svr *C4* plays a role in symptom production and virus movement, but it is not required for virus replication (57). Similarly, the replication of BCTVRepl-cGFP was not significantly affected by the *C4* gene in sugar beet and *N. benthamiana* (Fig. S6). However, mutation of the *C4* gene in BCTVRepl-cGFP reduced the HDR rate in tissues co-infiltrated with CLCB-nGFP. This may indicate that *C4* plays a role in HDR possibly by affecting the regulation of cell cycle-related genes (58) or by non-specifically binding to viral DNA and ds/ssDNA (59), thereby facilitating DNA recombination.

Mutation of *C3* reduced the number of GFP cells in tissues co-infiltrated with CLCB-nGFP and BCTVRepl-cGFP *C3m*, which correlated with DNA accumulation in *C3m* compared to the wild type. This suggests that *C3* may not have an independent role in promoting HDR between these two components. Consistent with the role of *C3* as a replication enhancer in other bipartite geminiviruses, mutation of BCTV *C3* was found to reduce viral DNA accumulation by three- to fourfold in protoplast cells (55). Finally, the potential role of virion-sense genes (*V1*, *V2*, and *V3*) in promoting DNA recombination in BCTV, either directly or through interaction with other viral proteins, cannot be ruled out. Therefore, the potential of these genes to influence viral recombination requires further investigation.

In conclusion, a model has been developed to facilitate the study of DNA recombination between BCTV and CLCB. This model enables the analysis of both viral and potential host factors that are involved in viral DNA recombination. Furthermore, this model can be applied to other geminiviruses. A similar approach has been successfully used to characterize the genetic requirements for homologous recombination in *Autographa californica*, a nucleopolyhedrovirus (46). Utilizing this model, we have demonstrated that *C2* and potentially *C4* play a direct role in HDR, as evidenced by the observation that mutation of *C2* and *C4* led to a significant reduction in HDR events, with minimal effect on the DNA accumulation. These genes can be evaluated for the improvement of genome editing by increasing the efficacy of the BCTV replicon for HDR using the CRISPR/CAS system. Furthermore, the inverted repeats and stem-loop motifs in the BCTV LIR were found to be essential for recombination between BCTVRepl-cGFP and the T-DNA construct, LnGFP. Mutation of stem-loop sequences through deletion or conversion further supported the possible role of Rep and structural elements of the BCTV LIR in DNA recombination.

## Data Availability

The nCATS sequence data are available upon request.

## SUPPLEMENTAL MATERIAL

Supplemental figures jvi.00016-25-s0001.pdf Figures S1 to S6.

Supplemental tables jvi.00016-25-s0002.docx Tables S1 and S2.

ASM does not own the copyrights to Supplemental Material that may be linked to, or accessed through, an article. The authors have granted ASM a non-exclusive, world-wide license to publish the Supplemental Material files. Please contact the corresponding author directly for reuse.

## References

[1] Wu M, Wei H, Tan H, Pan S, Liu Q, Bejarano ER, Lozano-Durán R. 2021. Plant DNA polymerases α and δ mediate replication of geminiviruses. Nat Commun 12:2780. doi:10.1038/s41467-021-23013-2 33986276 PMC8119979

[2] Saunders K, Lucy A, Stanley J. 1991. DNA forms of the geminivirus African cassava mosaic virus consistent with a rolling circle mechanism of replication. Nucleic Acids Res 19:2325–2330. doi:10.1093/nar/19.9.2325 2041773 PMC329438

[3] Jeske H, Lütgemeier M, Preiss W. 2001. DNA forms indicate rolling circle and recombination-dependent replication of Abutilon mosaic virus. EMBO J 20:6158–6167. doi:10.1093/emboj/20.21.6158 11689455 PMC125697

[4] Bonnamy M, Blanc S, Michalakis Y. 2023. Replication mechanisms of circular ssDNA plant viruses and their potential implication in viral gene expression regulation. MBio:e01692-23. doi:10.1128/mbio.01692-23 37695133 PMC10653810

[5] Preiss W, Jeske H. 2003. Multitasking in replication is common among geminiviruses. J Virol 77:2972–2980. doi:10.1128/jvi.77.5.2972-2980.2003 12584322 PMC149778

[6] Hanley-Bowdoin L, Bejarano ER, Robertson D, Mansoor S. 2013. Geminiviruses: masters at redirecting and reprogramming plant processes. Nat Rev Microbiol 11:777–788. doi:10.1038/nrmicro3117 24100361

[7] Richter KS, Kleinow T, Jeske H. 2014. Somatic homologous recombination in plants is promoted by a geminivirus in a tissue-selective manner. Virology (Auckl) 452–453:287–296. doi:10.1016/j.virol.2014.01.024 24606706

[8] Ascencio-Ibáñez JT, Sozzani R, Lee T-J, Chu T-M, Wolfinger RD, Cella R, Hanley-Bowdoin L. 2008. Global analysis of Arabidopsis gene expression uncovers a complex array of changes impacting pathogen response and cell cycle during geminivirus infection. Plant Physiol 148:436–454. doi:10.1104/pp.108.121038 18650403 PMC2528102

[9] Zerbini FM, Briddon RW, Idris A, Martin DP, Moriones E, Navas-Castillo J, Rivera-Bustamante R, Roumagnac P, Varsani A Ictv Report Consortium 2017. ICTV virus taxonomy profile: geminiviridae. J Gen Virol 98:131–133. doi:10.1099/jgv.0.000738 28284245 PMC5802298

[10] Poornima Priyadarshini CG, Ambika MV, Tippeswamy R, Savithri HS. 2011. Functional characterization of coat protein and V2 involved in cell to cell movement of cotton leaf curl Kokhran virus-Dabawali. PLoS ONE 6:e26929. doi:10.1371/journal.pone.0026929 22110597 PMC3217939

[11] Hormuzdi SG, Bisaro DM. 1993. Genetic analysis of beet curly top virus: evidence for three virion sense genes involved in movement and regulation of single- and double-stranded DNA levels. Virology (Auckl) 193:900–909. doi:10.1006/viro.1993.1199 8460493

[12] Nash TE, Dallas MB, Reyes MI, Buhrman GK, Ascencio-Ibañez JT, Hanley-Bowdoin L. 2011. Functional analysis of a novel motif conserved across geminivirus Rep proteins. J Virol 85:1182–1192. doi:10.1128/JVI.02143-10 21084480 PMC3020519

[13] Shakir S, Mubin M, Nahid N, Serfraz S, Qureshi MA, Lee TK, Liaqat I, Lee S, Nawaz-Ul-Rehman MS. 2023. REPercussions: how geminiviruses recruit host factors for replication. Front Microbiol 14:1224221. doi:10.3389/fmicb.2023.1224221 37799604 PMC10548238

[14] Zhang Z, Chen H, Huang X, Xia R, Zhao Q, Lai J, Teng K, Li Y, Liang L, Du Q, Zhou X, Guo H, Xie Q. 2011. BSCTV C2 attenuates the degradation of SAMDC1 to suppress DNA methylation-mediated gene silencing in Arabidopsis. Plant Cell 23:273–288. doi:10.1105/tpc.110.081695 21245466 PMC3051253

[15] Stenger DC, Carbonaro D, Duffus JE. 1990. Genomic characterization of phenotypic variants of beet curly top virus. J Gen Virol 71 (Pt 10):2211–2215. doi:10.1099/0022-1317-71-10-2211 2230726

[16] Stenger DC. 1994. Complete nucleotide sequence of the hypervirulent CFH strain of beet curly top virus. Mol Plant Microbe Interact 7:154–157. doi:10.1094/mpmi-7-0154 8167369

[17] Laufs J, Traut W, Heyraud F, Matzeit V, Rogers SG, Schell J, Gronenborn B. 1995. In vitro cleavage and joining at the viral origin of replication by the replication initiator protein of tomato yellow leaf curl virus. Proc Natl Acad Sci USA 92:3879–3883. doi:10.1073/pnas.92.9.3879 7732000 PMC42065

[18] Brown JK, Fauquet CM, Briddon RW, Zerbini M, Moriones E, Navas Castillo J. 2012. Geminiviridae, p 351–373. In King AMQ, Adams MJ, Carstens EB, Lefkowitz EJ (ed), Virus taxonomy: Ninth Report of the International Committee on Taxonomy of Viruses Elsevier. London.

[19] Choi I-R, Stenger DC. 1996. The strain-specific cis-acting element of beet curly top geminivirus DNA replication maps to the directly repeated motif of the ori. Virology (Auckl) 226:122–126. doi:10.1006/viro.1996.0634 8941329

[20] Zhou X. 2013. Advances in understanding begomovirus satellites. Annu Rev Phytopathol 51:357–381. doi:10.1146/annurev-phyto-082712-102234 23915133

[21] Briddon RW, Stanley J. 2006. Subviral agents associated with plant single-stranded DNA viruses. Virology (Auckl) 344:198–210. doi:10.1016/j.virol.2005.09.042 16364750

[22] Saeed M, Behjatnia SAA, Mansoor S, Zafar Y, Hasnain S, Rezaian MA. 2005. A single complementary-sense transcript of a geminiviral DNA beta satellite is determinant of pathogenicity. Mol Plant Microbe Interact 18:7–14. doi:10.1094/MPMI-18-0007 15672813

[23] Eini O, Dogra SC, Dry IB, Randles JW. 2012. Silencing suppressor activity of a begomovirus DNA β encoded protein and its effect on heterologous helper virus replication. Virus Res 167:97–101. doi:10.1016/j.virusres.2012.03.012 22504338

[24] Briddon RW, Bull SE, Amin I, Idris AM, Mansoor S, Bedford ID, Dhawan P, Rishi N, Siwatch SS, Abdel-Salam AM, Brown JK, Zafar Y, Markham PG. 2003. Diversity of DNA beta, a satellite molecule associated with some monopartite begomoviruses. Virology (Auckl) 312:106–121. doi:10.1016/s0042-6822(03)00200-9 12890625

[25] Saunders K, Bedford ID, Briddon RW, Markham PG, Wong SM, Stanley J. 2000. A unique virus complex causes Ageratum yellow vein disease . Proc Natl Acad Sci USA 97:6890–6895. doi:10.1073/pnas.97.12.6890 10841581 PMC18771

[26] Kharazmi S, Behjatnia SAA, Hamzehzarghani H, Niazi A. 2012. Cotton leaf curl Multan betasatellite as a plant gene delivery vector trans-activated by taxonomically diverse geminiviruses. Arch Virol 157:1269–1279. doi:10.1007/s00705-012-1290-2 22476203

[27] Fiallo-Olivé E, Navas-Castillo J. 2023. The role of extensive recombination in the evolution of geminiviruses, p 139–166. In Domingo E, Schuster P, Elena SF, Perales C (ed), Viral Fitness and Evolution: Population Dynamics and Adaptive Mechanisms doi:10.1007/978-3-031-15640-3_4. Springer International Publishing, Cham. 10.1007/978-3-031-15640-3_4 36592245

[28] Baltes NJ, Gil-Humanes J, Cermak T, Atkins PA, Voytas DF. 2014. DNA replicons for plant genome engineering. Plant Cell 26:151–163. doi:10.1105/tpc.113.119792 24443519 PMC3963565

[29] Čermák T, Baltes NJ, Čegan R, Zhang Y, Voytas DF. 2015. High-frequency, precise modification of the tomato genome. Genome Biol 16:232. doi:10.1186/s13059-015-0796-9 26541286 PMC4635538

[30] Dahan-Meir T, Filler-Hayut S, Melamed-Bessudo C, Bocobza S, Czosnek H, Aharoni A, Levy AA. 2018. Efficient in planta gene targeting in tomato using geminiviral replicons and the CRISPR/Cas9 system. Plant J 95:5–16. doi:10.1111/tpj.13932 29668111

[31] de Pater S, Klemann BJPM, Hooykaas PJJ. 2018. True gene-targeting events by CRISPR/Cas-induced DSB repair of the PPO locus with an ectopically integrated repair template. Sci Rep 8:3338. doi:10.1038/s41598-018-21697-z 29463822 PMC5820285

[32] Eini O, Schumann N, Niessen M, Varrelmann M. 2022. Targeted mutagenesis in plants using Beet curly top virus for efficient delivery of CRISPR/Cas12a components. N Biotechnol 67:1–11. doi:10.1016/j.nbt.2021.12.002 34896246

[33] Lefeuvre P, Moriones E. 2015. Recombination as a motor of host switches and virus emergence: geminiviruses as case studies. Curr Opin Virol 10:14–19. doi:10.1016/j.coviro.2014.12.005 25559880

[34] Varsani A, Martin DP, Navas-Castillo J, Moriones E, Hernández-Zepeda C, Idris A, Murilo Zerbini F, Brown JK. 2014. Revisiting the classification of curtoviruses based on genome-wide pairwise identity. Arch Virol 159:1873–1882. doi:10.1007/s00705-014-1982-x 24463952

[35] Strausbaugh CA, Eujayl IA, Wintermantel WM. 2017. Beet curly top virus strains associated with sugar beet in Idaho, Oregon, and a Western U.S. collection. Plant Dis 101:1373–1382. doi:10.1094/PDIS-03-17-0381-RE 30678603

[36] Martin DP, Biagini P, Lefeuvre P, Golden M, Roumagnac P, Varsani A. 2011. Recombination in eukaryotic single stranded DNA viruses. Viruses 3:1699–1738. doi:10.3390/v3091699 21994803 PMC3187698

[37] García-Andrés S, Tomás DM, Sánchez-Campos S, Navas-Castillo J, Moriones E. 2007. Frequent occurrence of recombinants in mixed infections of tomato yellow leaf curl disease-associated begomoviruses. Virology (Auckl) 365:210–219. doi:10.1016/j.virol.2007.03.045 17467025

[38] Gibson DG, Young L, Chuang R-Y, Venter JC, Hutchison CA, Smith HO. 2009. Enzymatic assembly of DNA molecules up to several hundred kilobases. Nat Methods 6:343–345. doi:10.1038/nmeth.1318 19363495

[39] Rouhibakhsh A, Priya J, Periasamy M, Haq QMI, Malathi VG. 2008. An improved DNA isolation method and PCR protocol for efficient detection of multicomponents of begomovirus in legumes. J Virol Methods 147:37–42. doi:10.1016/j.jviromet.2007.08.004 17870189

[40] Eini O, Benjes K, Dietrich K, Reichelt M, Schumann N, Varrelmann M. 2024. Insights into the molecular basis of beet curly top resistance in sugar beet through a transcriptomic approach at the early stage of symptom development. J Gen Virol 105:002026. doi:10.1099/jgv.0.002026 39311862

[41] Liu D, Shi L, Han C, Yu J, Li D, Zhang Y. 2012. Validation of reference genes for gene expression studies in virus-infected Nicotiana benthamiana using quantitative real-time PCR. PLoS ONE 7:e46451. doi:10.1371/journal.pone.0046451 23029521 PMC3460881

[42] Tyurin AA, Kabardaeva KV, Berestovoy MA, Sidorchuk YuV, Fomenkov AA, Nosov AV, Goldenkova-Pavlova IV. 2017. Simple and reliable system for transient gene expression for the characteristic signal sequences and the estimation of the localization of target protein in plant cell. Russ J Plant Physiol 64:672–679. doi:10.1134/S1021443717040173 PMC8203870 34179266

[43] Hajizadeh HS, Heidari B, Bertoldo G, Della Lucia MC, Magro F, Broccanello C, Baglieri A, Puglisi I, Squartini A, Campagna G, Concheri G, Nardi S, Stevanato P. 2019. Expression profiling of candidate genes in sugar beet leaves treated with leonardite-based biostimulant. High Throughput 8:18. doi:10.3390/ht8040018 31614507 PMC6970231

[44] Gilpatrick T, Lee I, Graham JE, Raimondeau E, Bowen R, Heron A, Downs B, Sukumar S, Sedlazeck FJ, Timp W. 2020. Targeted nanopore sequencing with Cas9-guided adapter ligation. Nat Biotechnol 38:433–438. doi:10.1038/s41587-020-0407-5 32042167 PMC7145730

[45] De Coster W, Weissensteiner MH, Sedlazeck FJ. 2021. Towards population-scale long-read sequencing. Nat Rev Genet 22:572–587. doi:10.1038/s41576-021-00367-3 34050336 PMC8161719

[46] Behjatnia SAA, Dry IB, Rezaian MA. 2001. Sequence divergence in new strains of Tomato leaf curl virus resulting in replication specificity. Austral Plant Pathol 30:337. doi:10.1071/AP01042

[47] Crouch EA, Passarelli AL. 2002. Genetic requirements for homologous recombination in Autographa californica nucleopolyhedrovirus. J Virol 76:9323–9334. doi:10.1128/jvi.76.18.9323-9334.2002 12186915 PMC136457

[48] Lin B, Akbar Behjatnia SA, Dry IB, Randles JW, Rezaian MA. 2003. High-affinity Rep-binding is not required for the replication of a geminivirus DNA and its satellite. Virology (Auckl) 305:353–363. doi:10.1006/viro.2002.1671 12573580

[49] Saunders K, Stanley J. 1999. A nanovirus-like DNA component associated with yellow vein disease of Ageratum conyzoides: evidence for interfamilial recombination between plant DNA viruses. Virology (Auckl) 264:142–152. doi:10.1006/viro.1999.9948 10544139

[50] Tao X, Zhou X. 2008. Pathogenicity of a naturally occurring recombinant DNA satellite associated with tomato yellow leaf curl China virus. J Gen Virol 89:306–311. doi:10.1099/vir.0.83388-0 18089755

[51] Shuja MN, Tahir M, Briddon RW. 2017. Occurrence of a recombinant molecule carrying sequences derived from an alphasatellite and the helper virus in cotton affected with cotton leaf curl disease. Trop plant pathol 42:397–402. doi:10.1007/s40858-017-0161-5

[52] Saunders K, Bedford ID, Stanley J. 2001. Pathogenicity of a natural recombinant associated with ageratum yellow vein disease: implications for geminivirus evolution and disease aetiology. Virology (Auckl) 282:38–47. doi:10.1006/viro.2000.0832 11259188

[53] George B, Alam CM, Kumar RV, Gnanasekaran P, Chakraborty S. 2015. Potential linkage between compound microsatellites and recombination in geminiviruses: evidence from comparative analysis. Virology (Auckl) 482:41–50. doi:10.1016/j.virol.2015.03.003 25817404

[54] Gonçalves MAFV, van Nierop GP, Holkers M, de Vries AAF. 2012. Concerted nicking of donor and chromosomal acceptor DNA promotes homology-directed gene targeting in human cells. Nucleic Acids Res 40:3443–3455. doi:10.1093/nar/gkr1234 22189101 PMC3333848

[55] Caracuel Z, Lozano‐Durán R, Huguet S, Arroyo‐Mateos M, Rodríguez‐Negrete EA, Bejarano ER. 2012. C2 from Beet curly top virus promotes a cell environment suitable for efficient replication of geminiviruses, providing a novel mechanism of viral synergism . New Phytologist 194:846–858. doi:10.1111/j.1469-8137.2012.04080.x 22404507

[56] Hormuzdi SG, Bisaro DM. 1995. Genetic analysis of beet curly top virus: examination of the roles of L2 and L3 genes in viral pathogenesis. Virology (Auckl) 206:1044–1054. doi:10.1006/viro.1995.1027 7856079

[57] Krenz B, Deuschle K, Deigner T, Unseld S, Kepp G, Wege C, Kleinow T, Jeske H. 2015. Early function of the Abutilon mosaic virus AC2 gene as a replication brake. J Virol 89:3683–3699. doi:10.1128/JVI.03491-14 25589661 PMC4403429

[58] Teng K, Chen H, Lai J, Zhang Z, Fang Y, Xia R, Zhou X, Guo H, Xie Q. 2010. Involvement of C4 protein of beet severe curly top virus (family Geminiviridae) in virus movement. PLoS One 5:e11280. doi:10.1371/journal.pone.0011280 20585583 PMC2892029

[59] Park J, Hwang H-S, Buckley KJ, Park J-B, Auh C-K, Kim D-G, Lee S, Davis KR. 2010. C4 protein of Beet severe curly top virus is a pathomorphogenetic factor in Arabidopsis. Plant Cell Rep 29:1377–1389. doi:10.1007/s00299-010-0923-8 20960205

