# Supplementary material for: Role of complementary-sense genes and intergenic region of beet curly top virus in intermolecular recombination frequency upon local infection in plants
Source: J Virol. 2025 Jul 8;99(8):e00016-25. doi: 10.1128/jvi.00016-25 (PMC12363183; doi:10.1128/jvi.00016-25)
Supplement: Supplemental figures — Figures S1 to S6. [file jvi.00016-25-s0001.pdf]

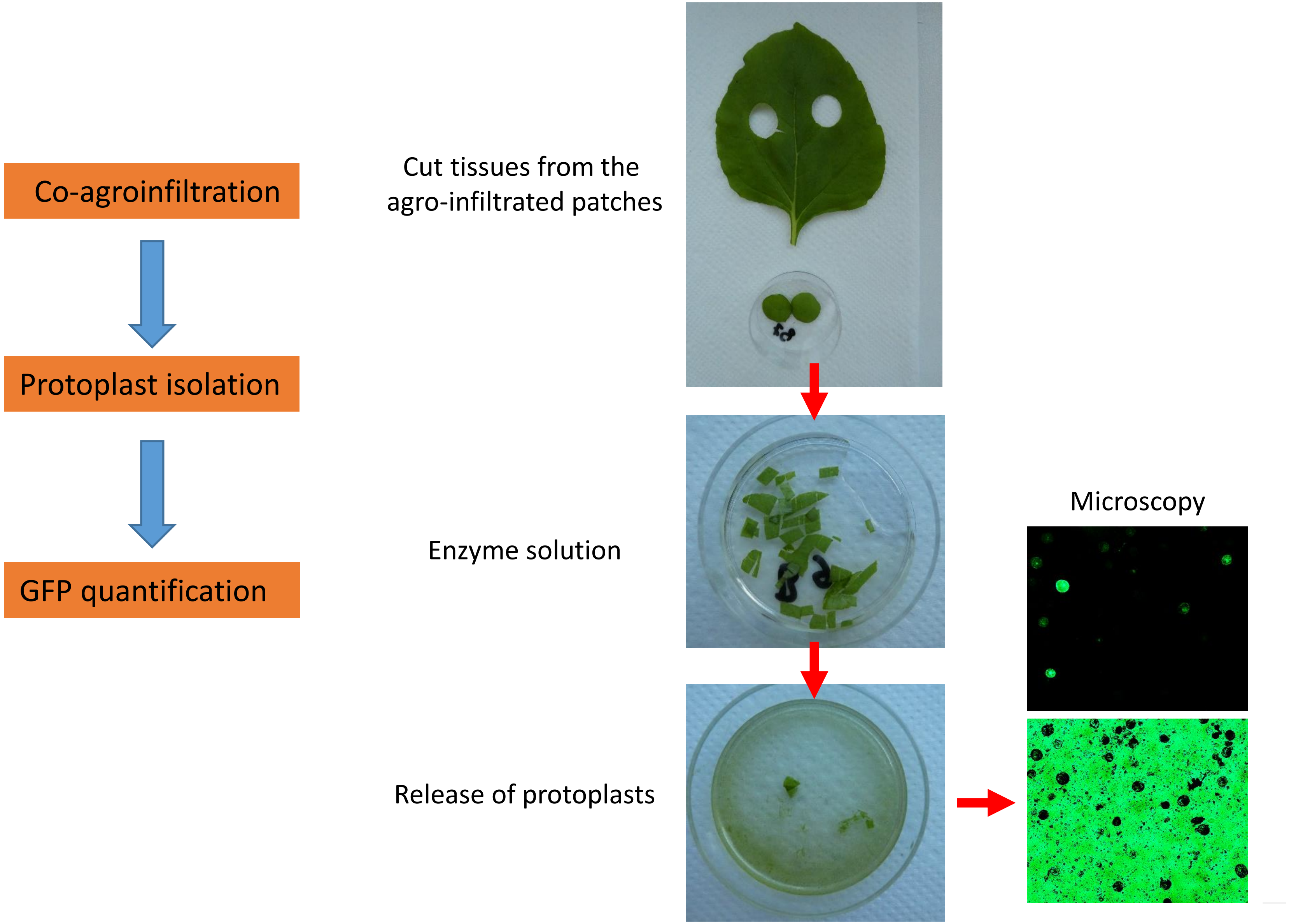

Fig. S1. Protoplast isolation and GFP monitoring in co-infiltrated *Nicotiana benthamiana* leaves. Co-infiltrated leaf patches with BCTVRepl-cGFP and LnGFP were cut and minced with a scalpel, then digested with enzyme solutions for 16 h at 22°C. Protoplast cells were collected by filtration and centrifugation (100 g for 5 min) and observed under UV microscope with GFP filter. The ratio of GFP cells to the total number of cells was recorded.

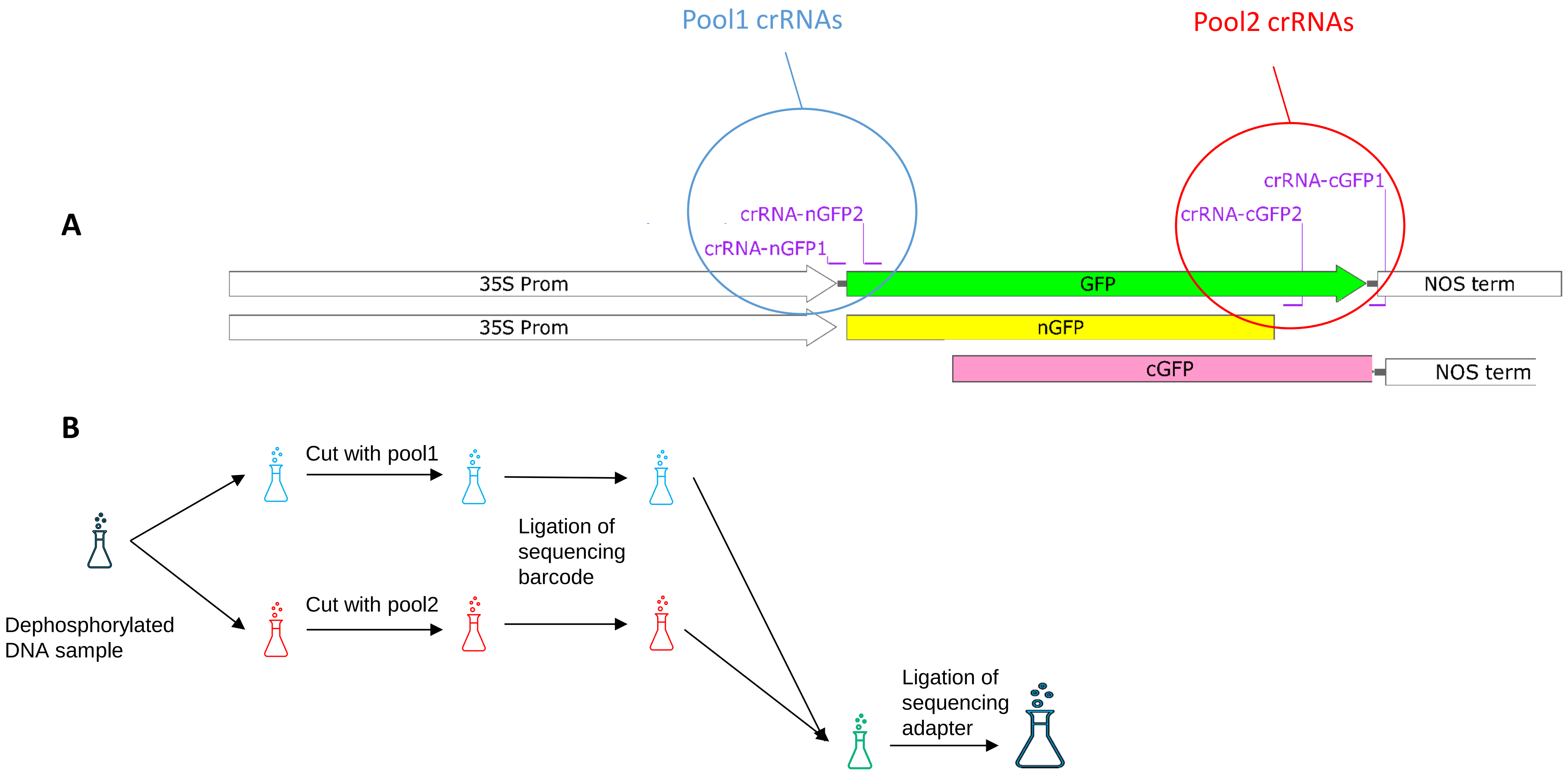

Fig. S2. Nanopore Cas12a targeted sequencing (nCATS) for the target GFP gene in leaf tissues co-infiltrated with BCTVRepl-cGFP and CLCB-nGFP. (A) Schematic map for the target GFP gene showing the position of four crRNAs used in this assay. (B) Steps of DNA sample preparation for nCATS sequencing including dephosphorylation, sequence-specific cutting with Cas12a ribonucleoproteins, sequencing barcode ligation, and sequencing adapter ligation.

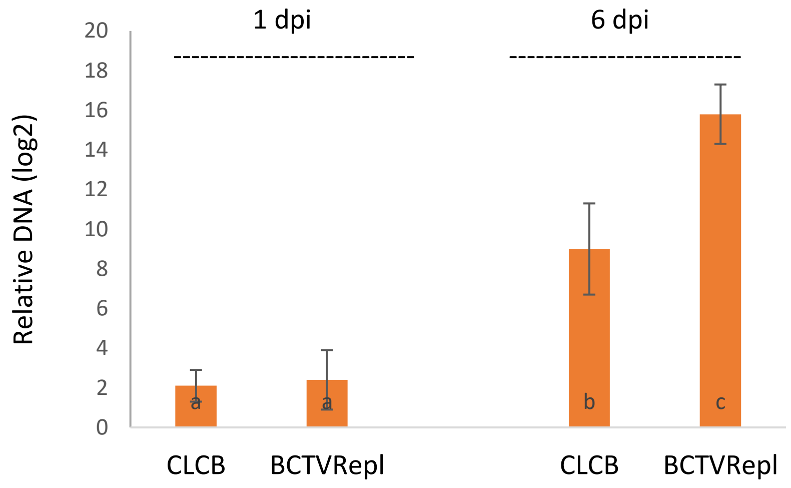

Fig. S3. DNA accumulation for the BCTV- and CLCB-derived replicons measured by real-time PCR. The CLCB construct was co-inoculated into *N. benthamiana* leaves. Total DNA was extracted at 1 and 6 days after inoculation and tested for accumulation of BCTVRepl and CLCB. Three biological replicates were tested by qPCR for each treatment and normalized to the plant housekeeping gene, F-box. Error bars are standard deviations. Bars with the same letter indicate statistically not significant differnces ($P < 0.01$).

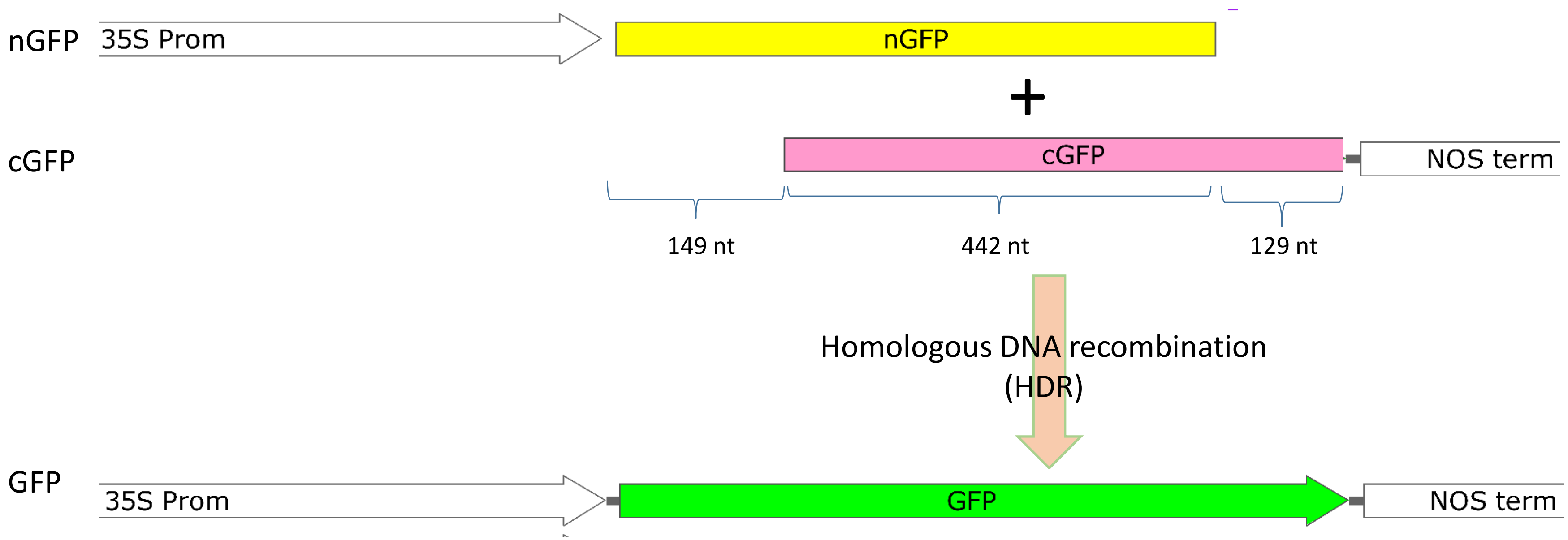

Fig. S4. Schematic maps for GFP recombination assay. N-terminal GFP contains the 35S promoter and the N-terminal of the GFP sequence (591 nt), cGFP contains the C-terminal of the GFP sequence (571 nt) and the NOS terminator. After recombination between nGFP and cGFP, a complete GFP sequence with visible phenotype appears.

A

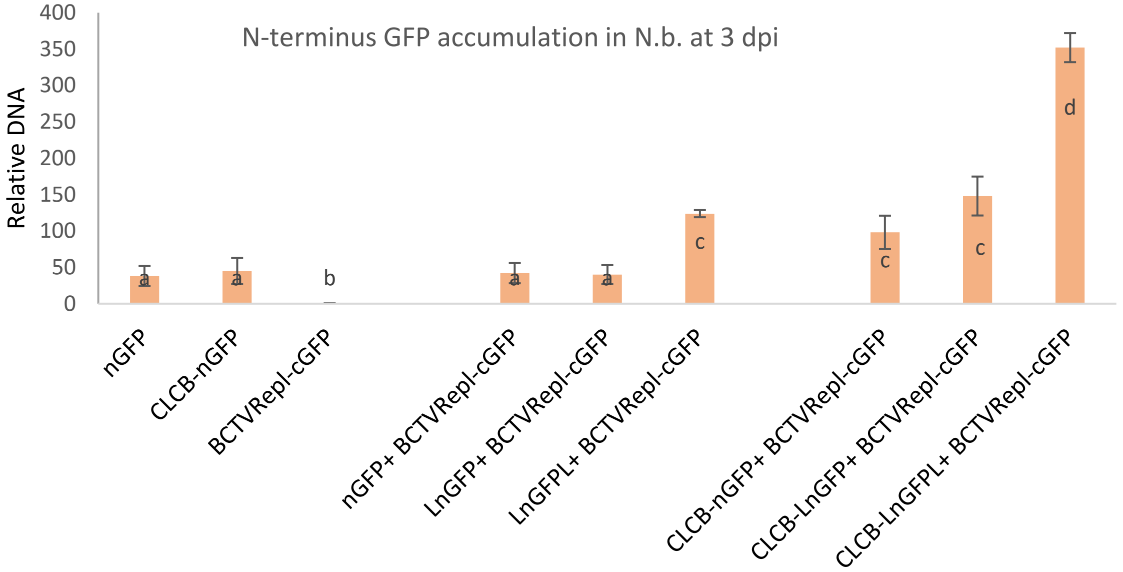

B

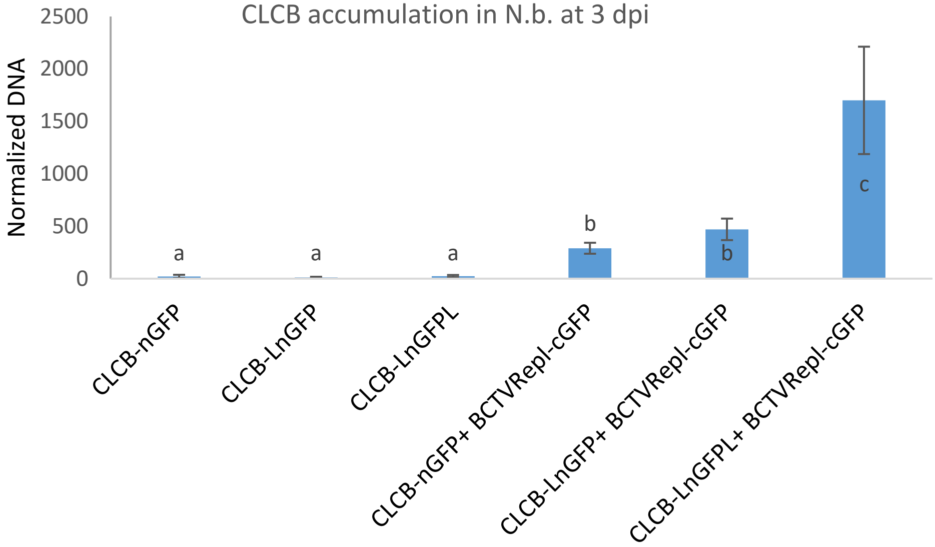

Fig. S5. Real-time PCR shows the accumulation of N-terminal GFP (A) and CLCB (B) in leaf tissues at 3 dpi. N-terminal GFP was specifically quantified in tissues co-infiltrated with BCTVRepl-cGFP and T-DNA (nGFP, LnGFP or LnGFPL) or CLCB constructs (CLCB-nGFP, CLCB-LnGFP or CLCB-LnGFPL). For control, leaf tissues were infiltrated with a single construct of nGFP, CLCB-nGFP or BCTVRepl-cGFP. CLCB was specifically quantified in the same co-infiltrated tissues. Control tissues were infiltrated with only CLCB-nGFP, CLCB-LnGFP or CLCB-LnLGFP. DNA templates were digested with *Dpn*I restriction enzyme before the qPCR assays. For each treatment, three biological replicates were tested and normalized to the housekeeping genes, F-box and GAPDH genes. Error bars indicate standard deviations. Bars with the same letter indicate statistically insignificant differences($P < 0.05$).

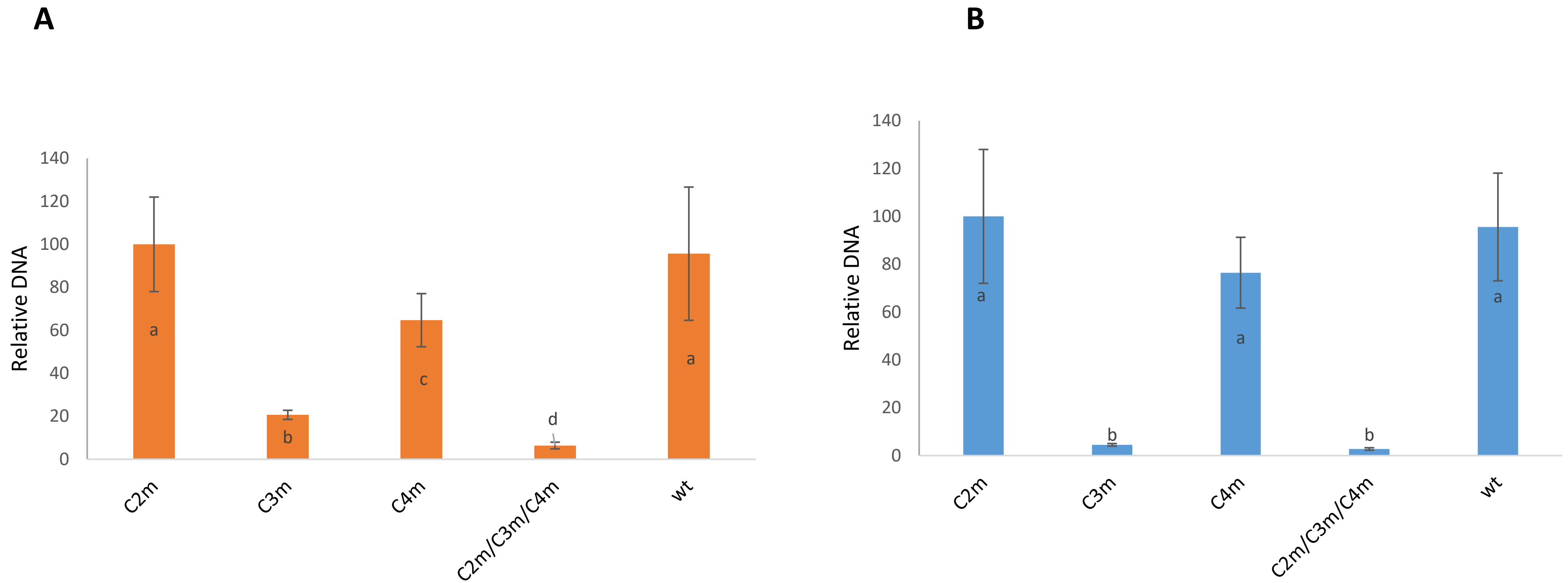

Fig. S6. Real-time PCR for DNA accumulation in BCTVRepl-cGFP mutants. Total DNA was extracted at 4 dpi from *N. benthamiana* (A) and *B. vulgaris* (B) tissues co-infiltrated with CLCB-nGFP and each of the BCTV-Repl-cGFP mutants (C2m, C3m, C4m, or C2m/C3m/C4m). As a control, wild-type BCTVRepl-cGFP (wt) was co-infiltrated with CLCB-nGFP. DNA templates were digested with *Dpn*I restriction enzyme before the qPCR assays. Three biological replicates were tested for each treatment by qPCR. QPCR data was normalized to the plant housekeeping genes, F-box and GAPDDH genes in N. benthamiana and sugar beet plants, respectively. For comparison, the DNA level in the wild-type BCTV-Repl-cGFP was adjusted to 100%. Bars with the same letter indicate statistically not significant differences (P<0.05). Error bars indicate standard deviation
