# Supplementary material for: Role of complementary-sense genes and intergenic region of beet curly top virus in intermolecular recombination frequency upon local infection in plants
Source: J Virol. 2025 Jul 8;99(8):e00016-25. doi: 10.1128/jvi.00016-25 (PMC12363183; doi:10.1128/jvi.00016-25)
Supplement: Supplemental tables — Tables S1 and S2. [file jvi.00016-25-s0002.docx]

Table S1. List of primers used in this study

| Primer name | Sequence (5'-3') | Target gene/genome | Application |
| --- | --- | --- | --- |
| CLCB-F | agtcgaattttccgacacg | CLCuMB | qPCR |
| CLCB-R | gtggtacctaccctcccag |  |  |
| pzf-CLCB-F | GAAACTTCACGATCGGCTCTAGAGGCCATGGagtcgaattttccgacacg | CLCuMB | CLCB clone |
| CLCMB-SpeI-R | atttccatcaaataagcagaaaACTAGTattccaaacacaaaccagca |  |  |
| CLCMB-SpeI-F | gcattgctggtttgtgtttggaatACTAGTTttctgcttatttgatggaaa |  |  |
| pzf-CLCB-R | CCCAAGATCTGGCCCTTAAGGCCTGTCGACattccaaacacaaaccagca |  |  |
| GFP-Spe-F | ccACTAGTtactggaaaactacctgttcc | 35S-GFP | BCTVRepl-cGFP clone |
| Nos-Spe-R | ccACTAGTgatctagtaacatagatgac |  |  |
| BS-LIR-Sma_F | tCCCGGGattgaatcgggctctcttc | BCTVRepl-GFP | BCTV-LnGF clone |
| GFP-Hind-R | gggAAGCTTggtaaaaggacagggccatc |  |  |
| BS-LIR-Hind-F | aaaAAGCTTattgaatcgggctctcttc | BCTV | CLCB-LnL-GF clone |
| BS-LIR-Hind-R | cccAAGCTTtttataagtacatatacatgt |  |  |
| BSLIRm-R | GTGGTATTTAAGAGttttCAAAGgcggCAATTGATAGG | LnGFP | LnGFP mut-l clone |
| BSLIRm-F1 | TTTGAAAACTCTTAAATACCACcaagg |  |  |
| BSLdel-F | ccatccgcatcggatggcccccaaaaaatac | LnGFP | LnGFP mut-d clone |
| BSLdel-R | tgggggccatccgatgcggatggccccttgg |  |  |
| BSCsm-F | aaCACATAGGAAgcattaatattaccggatggc | LnGFP | LnGFP mut-s clone |
| BSCsm-R | gcTTCCTATGTGttggtggtatttaagagcacc |  |  |
| pZF-R | CCGATTATTCTAATAAACGCTC | LnGFP | LnGFP mut clone |
| pZF-F | ACTTCACGATCGGCTCTAGA |  |  |
| BCTV-F3 | TAAACACCTGGCCCACATTGT | BCTV-Svr | qPCR |
| BCTV-R3 | TCAACCACCTTTTCCTCTTTCTTC |  |  |
| BC-F4 | ggACTAGTcggataatactaatatgtatg | BCTVRepl-GFP | BCTVRepl-cGFP C2m clone |
| BS-R2 | tGGGCCCGTTTAAACttataagtacatatacatgta |  |  |
| BCIR-C2m-F | atatgTtatgtagtgtggtctatatcTtatag |  |  |
| BCIR-C2m-R | ctataAgatatagaccacactacataAcatat |  |  |
| BCTV-F3 | TAAACACCTGGCCCACATTGT | BCTV | qPCR |
| BCTV-R3 | TCAACCACCTTTTCCTCTTTCTTC |  |  |
| 35S-s | CTATATAAGGAAGTTCATTTCATTTGGAGA | nGFP | qPCR |
| GFP-R4 | gtgcccattaacatcaccatc |  |  |

Table S2. CRISPR RNA (crRNA) sequences used in this study

| crRNA | Sequence (5'-3') | target motif |
| --- | --- | --- |
| crRNA-nGFP1 | tttggagagaacacgggggactcta | 35S promoter |
| crRNA-nGFP2 | tttcactggagttgtcccaattctt | N-terminal GFP |
| crRNA-cGFP1 | tttgaacgatcggggaaattcgagc | NOS terminal |
| crRNA-cGFP2 | tttcgaaagggcagattgtgtggacagg | C-terminal GFP |
